# Ganglioside GM1 slows down Aβ(1-42) aggregation by a primary nucleation inhibitory mechanism that is modulated by sphingomyelin and cholesterol

**DOI:** 10.1038/s42004-025-01846-y

**Published:** 2025-12-13

**Authors:** Nima Sasanian, Vesa Halipi, Mikaela Sjögren, Johannes Bengtsson, David Bernson, Elin K. Esbjörner

**Affiliations:** Division of Chemical Biology, Department of Life Sciences, Chalmers University of Technology, Kemivägen 10, S-412 96 Gothenburg, Sweden

**Keywords:** Protein aggregation, Protein aggregation, Biophysical chemistry

## Abstract

The conversion of soluble amyloid-β peptides into fibrils is central in Alzheimer’s disease. Lipids modulate amyloid-β aggregation, but whilst the mechanistic effect of individual lipid species is increasingly addressed, principles explaining their combinatorial contributions in biologically heterogenous membranes remain to be established. We used kinetic analyses to establish an inhibitory mechanism of GM1 gangliosides on the aggregation of amyloid-β variant Aβ(1-42) by which membrane-associated GM1 sequesters soluble Aβ(1-42) and retards primary nucleation. The kinetic inhibition increased in presence of the raft-enabling lipids cholesterol and sphingomyelin, although these lipids, intrinsically, catalysed primary and secondary nucleation respectively. These results decipher important trade-offs between the specific chemical properties of lipids and their general contributions to the physical state of membranes, show principles of competition, and identify low fluidity domains as key regulators of membrane-mediated Aβ(1-42) aggregation. The study thereby highlights a versatile, regulatory role of membranes in the molecular pathology of Alzheimer’s disease.

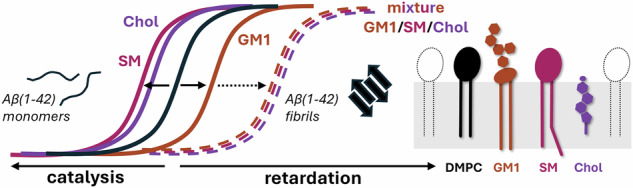

## Abstract (web summary)

Lipids can modulate amyloid-β aggregation, yet the combined effects of lipids in heterogeneous membranes remain unclear. Here, the authors reveal that ganglioside GM1 inhibits amyloid-β aggregation by sequestering soluble Aβ(1-42) and retards primary nucleation, with cholesterol and sphingomyelin modulating this effect, highlighting the mechanism of membrane-mediated Aβ(1-42) aggregation.

## Introduction

Alzheimer’s disease (AD) and other forms of dementia affect around 55 million people worldwide^1^. Dementia is currently the seventh leading cause of death and a major cause of disability and dependency among older people globally^2^. AD, which is the underlying cause of at least 60–70% of dementia cases, is pathologically linked to the aggregation and deposition of amyloid-β (Aβ) peptides into extracellular plaques^3^ as well as the formation of intraneuronal tau tangles^4^. Aβ aggregation is an early pathological feature of AD^5^ and has become an attractive therapeutic target^6^. Recent advances in this area has resulted in clinical approval of antibodies that target aggregated forms of Aβ and thereby moderately slow down the progression of early AD symptoms^7^. Better molecular and mechanistic understanding of the Aβ aggregation process and its modulation by intrinsic and extrinsic factors is, however, still needed to improve target recognition and efficacy of anti-aggregation treatments.

The brain is highly enriched in lipids^8^ and disruption to brain lipid homoeostasis and lipid membrane composition is a common pathological finding in AD^9,10^ as well as generally associated with aging^11^. Aβ peptides are likely to be affected by lipid alterations as they are produced and prevail in membrane-rich extra- and intracellular^12^ locations of the brain, such as neuronal synapses^13^, mitochondria^14^, the trans-Golgi network^15^, the endoplasmic reticulum (ER)^16^, and endolysosomes^17^. Membrane lipids are, furthermore, ubiquitously found within Aβ plaques^18^ suggesting that they may have important regulatory roles in Aβ-associated pathology^19^. In vitro biophysical studies have indeed shown that synthetic lipid vesicles^19–23^ as well as cell-derived extracellular vesicles^24^ can profoundly alter the rates and mechanisms of Aβ fibrillation. However, reported effects are diverse, ranging from catalytic in the case of for example phospholipids with choline head groups^23^ or cholesterol^21^ to inhibitory in presence of vesicles designed to mimic the membrane composition of Golgi and ER^20^. In some cases, such as for phosphatidylserine, there are conflicting reports of either catalytic^23^ or inhibitory^25^ effects. Furthermore, it has been suggested that Aβ interactions with cholesterol-rich membrane domains can both facilitate and hinder the formation of neurotoxic Aβ species^26–28^. This indicates that not only specific lipids but also their interplay and organisation within the lipid bilayer is important. However, the principles and mechanisms that determine the net aggregation-modulatory effects of complex biological membranes, where catalytic and inhibitory lipids inevitably co-exist, remains a challenge.

Sialylated glycosphingolipid gangliosides (GMs) are abundant in the brain^29^ and important in the regulation of neuronal physiology^30^. They have gained particular attention for their interactions with Aβ peptides and association with AD pathology^31^. The monosialotetrahexosylganglioside GM1 (GM1) has specifically been reported as down-regulated in temporal and frontal cortex regions of the AD brain^32^, has been found as a component of Aβ plaques^18^, and can form complexes with soluble Aβ peptides in the cerebral cortex^33^. Furthermore, GM1 has a preferential location to outer leaflets of neuronal plasma membranes, inner leaflets of endosomes, and to membrane vesicles released from neuronal cells, such as exosomes^30,34–36^, and therefore coincides with biological locations where Aβ peptides are abundant. In vitro studies have confirmed the formation of GM1-Aβ complexes^37,38^ associated them with the induction of Aβ secondary structure^39^, and shown that their formation can be potentiated upon GM1 clustering into lipid rafts^40^. However, the impact of GM1 on Aβ aggregation into amyloid fibrils is not entirely clear. Reports have proposed that GM1 can either inhibit^41^ or accelerate^42^ oligomer and fibril formation. This suggests that GM1 may be a context-dependent modulator of Aβ aggregation^43^ and motivates further exploration.

In this study, we have used bulk protein aggregation assays and modelling of kinetic data^44^ to explore how GM1-containing lipid membranes with complex lipid composition affects the rates and mechanisms of Aβ(1-42), addressing the role of GM1 as well as the existence of competitive and/or synergistic aggregation-modulatory effects. We show that membrane-associated GM1 delays the self-assembly of Aβ(1-42) into amyloid fibrils by interfering with the primary nucleation reaction step. Guided by this observation, we have further systematically explored how this GM1-mediated delay of Aβ(1-42) fibrillation is modulated by sphingomyelin (SM) and/or cholesterol (Chol), two other lipids with reported association to AD pathology^9,18,28^, which are, furthermore, together with GM1, known to engage in the formation of lipid rafts^40^. This allowed us to not only focus on the role of individual lipids, but to explore how both the chemical and physical complexity of a lipid bilayer can contribute to regulate membrane-mediated control of protein aggregation. We report that these lipids can have both synergistic and competitive effects on Aβ(1-42) fibrillation, depending on their combination and mixing ratio. Specifically, our data highlights the importance of membrane fluidity, and alterations thereof, in shaping a membrane’s aggregation-modulatory effect. Our study thereby conceptually expands current molecular and mechanistic understanding of how biological membranes modulate protein aggregation, addressing lipids that are specifically relevant in the context of Aβ pathology and AD.

## Results

### Lipid vesicles modulate Aβ(1-42) aggregation rates differently depending on their membrane compositions

**Fig. 1:**
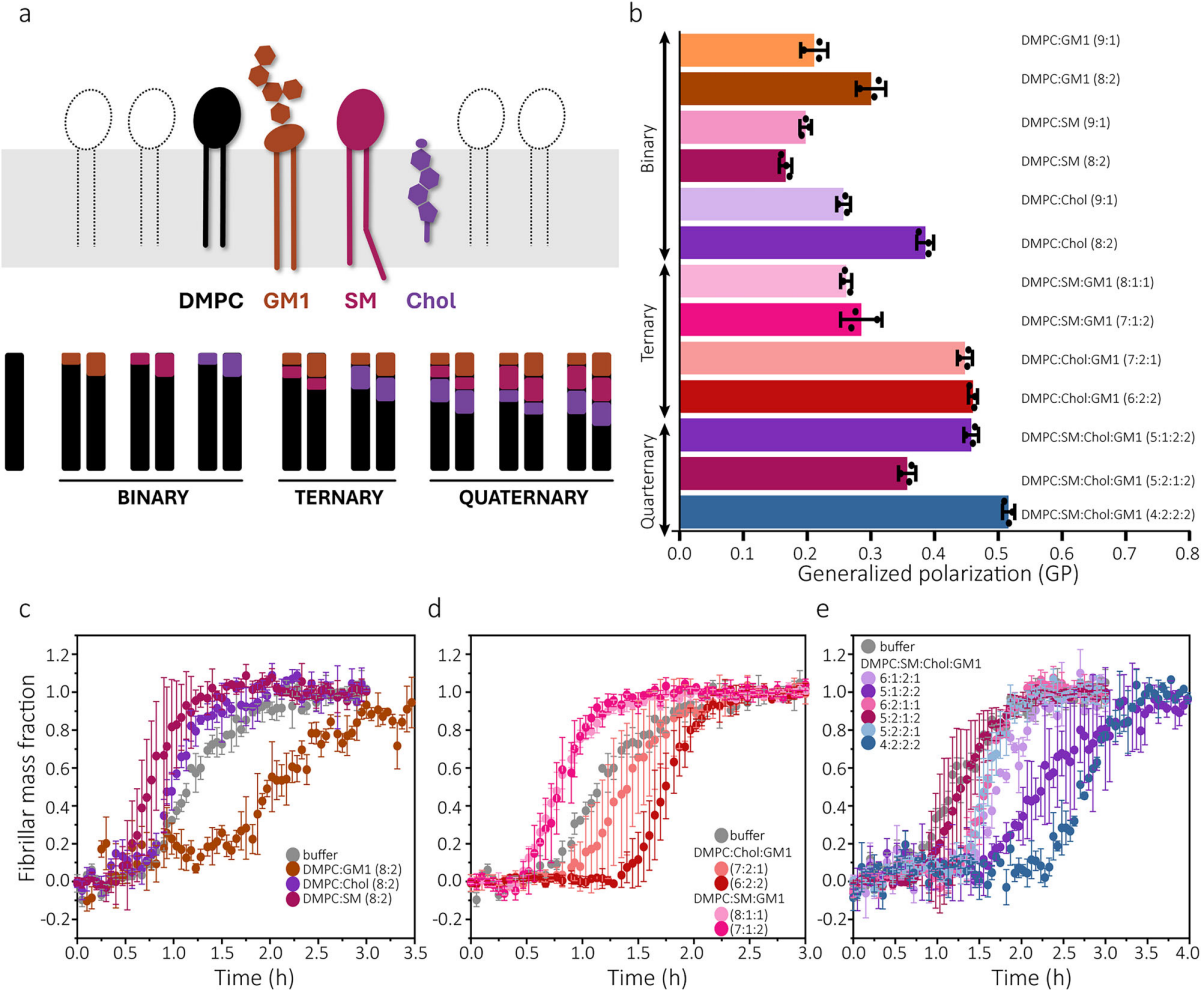
Lipid vesicles with different constituents and properties show diverse effects on Aβ(1-42) aggregation. **a** Schematic illustration of an idealised membrane with the lipids used in this study and a depiction of how they were mixed in different combinations to prepare large unilamellar vesicles (LUVs) with increasing lipid complexity. All LUVs had DMPC as the base lipid component and 0, 10, or 20 mol% of GM1, SM and/or Chol (see also Table S1). **b** Laurdan fluorescence (generalised polarisation, GP) of a subset of the LUVs recorded at 37 °C to compare their membrane fluidities. Aggregation kinetics of 2 μM size-exclusion chromatography purified Aβ(1-42) monomers in absence (buffer) or presence of 100 μM (total lipid concentration) of LUVs with **c** binary **d** ternary and **e** quaternary lipid compositions. The kinetics were monitored using thioflavin-T (ThT) fluorescence. Lipid molar ratios of the LUVs are indicated in each figure legend. All kinetic experiments were performed in technical triplicate (n = 3) and repeated (N = 3). The data in the figure are from one representative independent experiment, and the error bars represent standard deviation of the mean of three technical replicates (n = 3).

We prepared 20 different types of large unilamellar vesicles (LUVs; nominal diameter of 100 nm) with systematic variations in lipid content. The LUVs contained synthetic 1,2-dimyristoyl-*sn*-glycero-3-phosphocholine (DMPC), GM1, SM, and/or Chol (Fig. 1a, Supplementary Table 1). The GM1, SM, and Chol lipids were chosen because of their association with AD pathology^19^, but also because they are known to jointly participate in lipid domain formation (e.g., rafts)^40^. The zwitterionic phosphatidylcholine lipid DMPC (14:0) was included as a base phospholipid as it, as opposed to for example 1,2-dioleoyl-*sn*-glycero-3-phosphocholine (DOPC) (18:1)^21^ (Supplementary Fig. 2), does not affect Aβ(1-42) aggregation on its own^21^. The inertness of DMPC also in our setup was confirmed (Supplementary Fig. 3). Prior to the kinetic analyses, we used the laurdan assay^45^ to examine a subset of the LUVs with respect to their relative membrane fluidities (Fig. 1b, Supplementary Fig. 1). The observed laurdan values indicate membrane fluidities ranging from liquid disordered (generalised polarisation, GP, ~0.15) to liquid ordered (GP ~ 0.55)^46,47^.

The LUVs (0–100 µM concentration) were used in thioflavin-T (ThT) fluorescence monitored aggregation kinetic assays to study the aggregation of recombinant Aβ(1-42) monomers (2 μM). To ensure monomers as starting point of each reaction, Aβ(1-42) was subjected to size exclusion chromatography (SEC) immediately prior to each experiment (Supplementary Fig. 4) and monomers were collected from the appropriate elution volume^48^ as detailed in the Methods section. All kinetic experiments were performed in technical triplicate and repeated on at least three separate occasions (see Supplementary Fig. 5 on variability between repeats). The resulting, normalised, kinetic data are shown in Fig. 1c–e and Supplementary Fig. 6, whereas the end-point ThT values are reported in Supplementary Fig. 7.

**Fig. 2:**
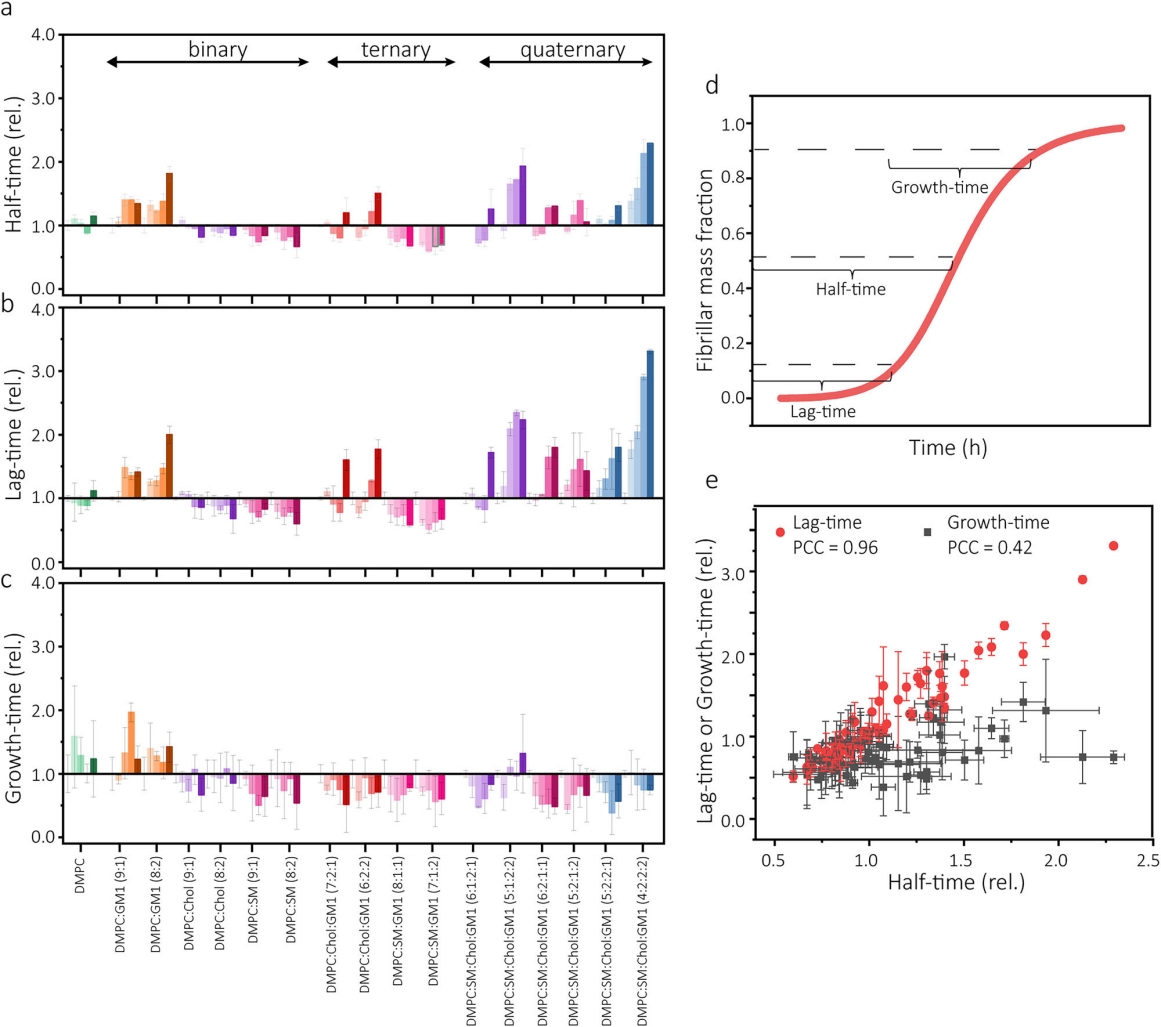
Effect of LUVs on different Aβ(1-42) aggregation kinetic parameters. Lipid-induced changes to the half-times (**a**) lag-times (**b**), and growth-times (**c**) of Aβ(1-42) aggregation reported relative to the kinetics in buffer (absence of large unilamellar vesicles (LUVs)). Each group of bars represent consecutively 2, 20, 50, and 100 μM (lipid equivalents) of the indicated LUV type. The data were extracted from the kinetic curves in Fig. S6 as defined in (**d**) and further described in the Methods section. **d** Schematic illustration of the definition of half-time, lag-time, and growth-time as plotted in (**a–c**). **e** Correlations of lag-times or growth-times with half-times for all data shown in (**a**–**c**), calculated with Pearson’s correlation coefficient (PCC). Error bars represent standard deviation of three technical replicates (n = 3).

Atomic force microscopy (AFM) images of mica-deposited samples taken at end-points of each aggregation reaction confirmed the formation of amyloid fibrils under all tested conditions. Significant quantities of soluble oligomeric species were not observed (Supplementary Figs. 8–11). The fibrils were about 0.9 μM long (Supplementary Fig. 12a) and 3 nm thick (Supplementary Fig. 12b) in all reactions, suggesting that the LUVs had little effect on the macroscopic appearances of the aggregates. By contrast, the LUVs had significant and diverse effects on Aβ(1-42)’s aggregation rate. We observed both catalysis and delay of fibril formation depending on the LUV composition (Fig. 1c–e, Supplementary Fig. 6), as further illustrated by calculations of the change in reaction half-times for Aβ(1-42) aggregation compared to the reaction half-time for Aβ(1-42) aggregation in buffer (Fig. 2a).

We first analysed LUVs with binary lipid compositions (DMPC:X). This allowed us to ascribe aggregation modulatory effects to GM1, SM, and Chol. GM1 increased aggregation half-times (Figs. 1c, 2a, Supplementary Fig. 6c and d) and thus slowed down Aβ(1-42) fibril formation whereas SM and Chol had catalytic effects (Fig. 1c, Fig. 2a, Supplementary Fig. 6a, b, e and f). We denote these as the intrinsic aggregation-modulatory effects of these lipids, although stringently it is their effects when present at relatively low molar ratios in a liquid disordered (Fig. 1b) DMPC bilayer. When the complexity of the LUVs was increased by combination of more lipids, we observed a wider variety of aggregation modulatory effects. They ranged from competitive between lipid species with opposing intrinsic effects to entire dominance by one lipid species (Figs. 1d and e, 2a, Supplementary Fig. 6g–p,) and suggest that biological membranes, can have diverse impacts and significant capacity to fine-tune Aβ(1-42) aggregation and solubility.

### GM1, cholesterol and SM lipids modulate different reaction steps in the Aβ(1-42) fibril assembly pathway

The LUVs altered both aggregation rates (Fig. 2a) and the shapes of the kinetic curves (Supplementary Fig. 6a–f), suggesting that their presence change the Aβ(1-42) fibril assembly pathway. Data simulations of amyloid formation have shown that inhibition of specific reaction steps manifests as distinctive alterations to kinetic curve shapes, such as extended lag-time and growth-time for primary and secondary nucleation inhibitors, respectively^49^. Fig. 2b and c show the lag-times and growth-time of the kinetic data, defined according to Fig. 2d and in the Methods section. We report strong correlation (Fig. 2e, Pearson’s correlation coefficient, PCC = 0.96) between changes in half-times and lag-times, suggesting that alterations in primary nucleation rates explain most of the observed lipid membrane induced changes to the kinetic data. Further, most of the LUVs decreased Aβ(1-42) aggregation growth-times (Fig. 2c), but the change in growth-time was largely invariant to LUV type and hence poorly correlated to changes in aggregation half-times (Fig. 2e, PCC = 0.42). A possible explanation to this observation is that lipid membranes may possess generic ability to catalyse secondary reaction steps, possibly via fibril interactions on the membrane surface^23^.

**Fig. 3:**
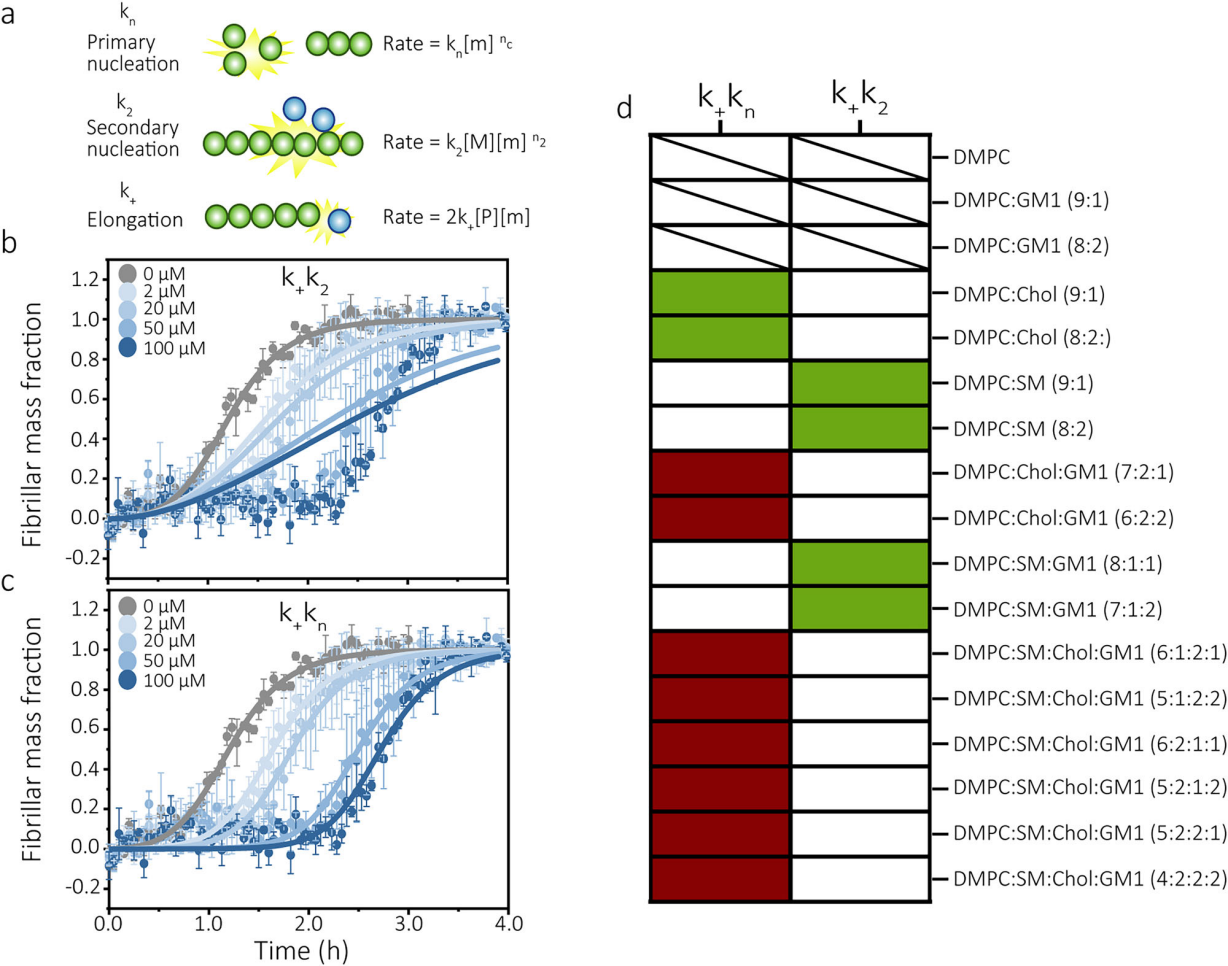
Analysis of Aβ(1-42) aggregation kinetic parameters. **a** Schematic illustration of key mechanistic steps in amyloid formation and their associated rate constants. The relations of the rate constants to the macroscopic aggregation rate are indicated by the equations to the right, where [m] is the monomer concentration, [M] is the fibril concentration in monomer units, and [P] is the fibril concentration in fibril units. n_c_ denotes the size (number of monomers) of the primary nuclei and n_2_ the size of the secondary nuclei. Examples of the fitting of a secondary nucleation dominated kinetic model with saturation to experimental data of Aβ(1-42) aggregation in presence of DMPC:SM:Chol:GM1 (4:2:2:2) LUVs keeping either k_+_k_2_ (**b**) or k_+_k_n_ (**c**) as the free parameter. Error bars represent standard deviation of three replicates. **d** Heat-map showing the best fitting kinetic model for each large unilamellar vesicle (LUV) type (e.g., if the change in aggregation rate is best described by variation in k_+_k_n_ (left column) or k_+_k_2_ (right column)) determined based on smallest mean residual error (MRE) (Table S2). Green and red indicates catalysis and delay of aggregation (e.g., increase or decrease of the indicated rate constant) respectively. Dashed squares indicate that the data could not be fitted by the model.

We further examined the mechanisms by which the LUVs modulated Aβ(1-42) aggregation by fitting rate laws of amyloid growth to the kinetic data. We used a secondary nucleation-dominant reaction model with saturation which has previously been used to describe Aβ(1-42) aggregation in buffer^50^. The model fits data to the compounded rate constants *k*_*+*_*k*_*n*_ and *k*_*+*_*k*_*2*_ (see Fig. 3a for definitions). We fitted the data twice, keeping either *k*_*+*_*k*_*n*_ or *k*_*+*_*k*_*2*_ as global constants (Fig. 3b and c, Supplementary Figs. 13–15, Supplementary Table 2) and explored how well the kinetic data could be explained by variation in the other rate constant. The dominant Aβ(1-42) aggregation-modulatory mechanism in presence of different LUVs was determined based on the best fit (i.e., lowest mean residual error, MRE) out of the two fittings (as described in Methods and shown in Supplementary Table 2). We found that most LUVs altered the kinetics of Aβ(1-42) aggregation in a way that is best described by variation in *k*_*+*_*k*_*n*_ and hence primary nucleation, consistent with the trends reported in Fig. 2. The kinetics in presence of some LUVs with SM content were, however, better described by changes in *k*_*+*_*k*_*2*_ (Fig. 3d).

### GM1 reduces primary nucleation, sequesters soluble Aβ(1-42) and abrogates fibril toxicity

**Fig. 4:**
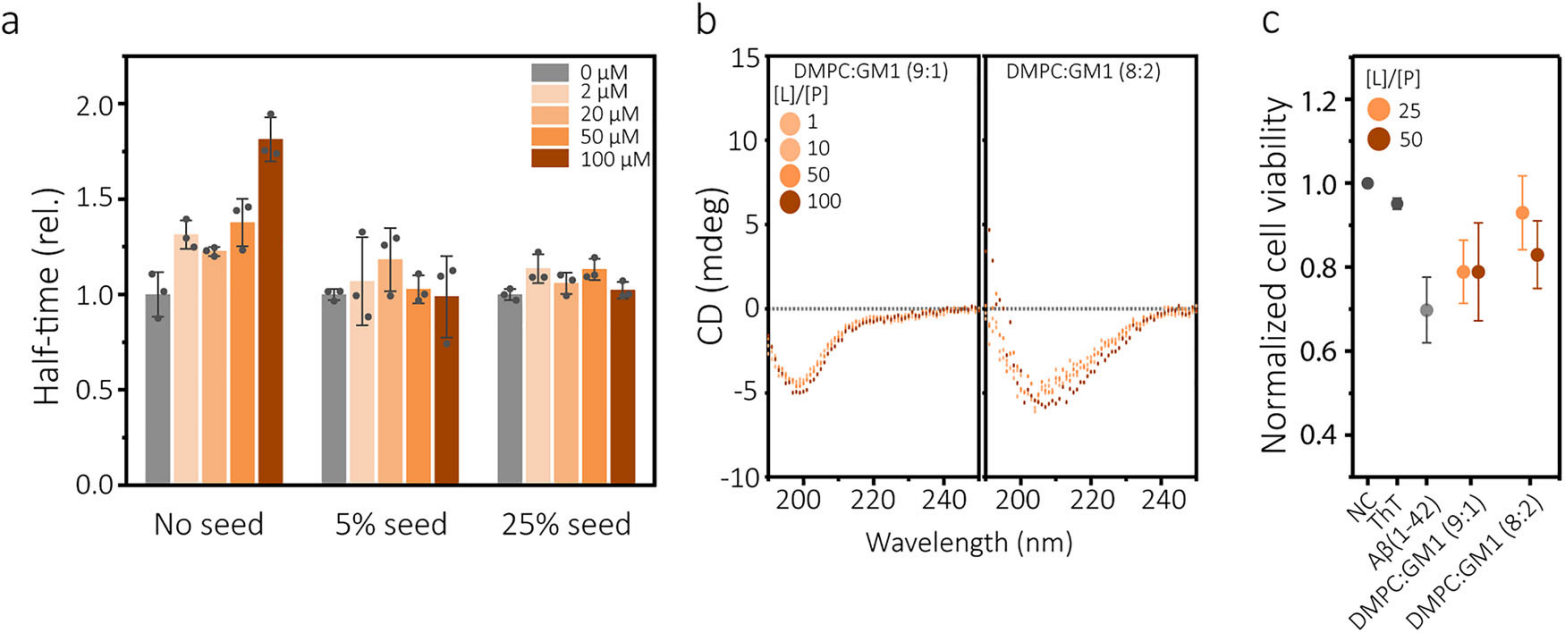
Analysis of the inhibitory GM1- Aβ(1-42) interaction. **a** Change in half-times of seeded Aβ(1-42) aggregation in presence of increasing concentrations of DMPC:GM1 (8:2) LUVs. Data are reported relative to the half-times of seeded Aβ(1-42) aggregation in buffer. The corresponding kinetic curves are shown in Fig. S6d and Fig. S16a and b. Error bars represent standard deviation of three replicates. **b** Circular dichroism (CD) spectra of 10 µM Aβ(1-42) monomer solutions recorded immediately upon addition of large unilamellar vesicles (LUVs) at a molar concentration ratio, [L]/[P] of 1, 10, 50, or 100. **c** Toxicity of samples collected at the aggregation end-point of Aβ(1-42) reactions in absence (buffer) or presence of DMPC:GM1 (9:1) and DMPC:GM1 (8:2) LUVs. The toxicity was estimated as cell viability following 24 h of treatment of SH-SY5Y cells with 1 µM of fibril solution and measured using the alamar blue metabolic assay. NC refers to negative control (buffer treatment) and ThT (thioflavin-T) to cell treatment with buffer containing the same amount of ThT (5 μM) as in the fibril samples. Error bars represent standard deviation of three technical replicates.

Given the many associations of GM1 with AD pathology, we decided to study this lipid more closely. Interestingly, it was not possible to fit the Aβ(1-42) aggregation kinetic data recorded in presence of binary mixture DMPC:GM1 LUVs (Fig. 3d) because of the appearance of a bimodal lag phase (Supplementary Fig. 6c and d). This suggests co-existence of two parallel inhibition processes operating on different timescales, possibly relating to the lateral organisation of GM1 within the DMPC bilayer. We therefore proceeded to carry out seeded aggregation kinetics experiments with different concentrations of pre-formed Aβ(1-42) fibrils as seeds (Supplementary Fig. 16a and b). With seeding, the DMPC:GM1 LUVs lost their inhibitory effect (Fig. 4a), which supports that GM1 is a kinetic inhibitor of primary nucleation. Using circular dichroism (CD) spectroscopy, we found evidence of interactions between  monomerized Aβ(1-42) (freshly prepared using SEC as described in Methods, Supplementary Fig. 4) and the LUVs. The interaction resulted in a slight increase  in negative ellipticity in the 210–220 nm region of the CD spectra. The interaction was stronger in LUVs with 20 mol% GM1, and the CD spectral change suggests induction of β-sheet structure, consistent with published solid-state NMR data^51^. Altogether, this suggests that membrane-associated GM1 sequesters soluble (monomeric or oligomeric) Aβ(1-42) and that the delay in aggregation could be related to a lowering of the concentration of aggregation-accessible peptides in solution. We further found that Aβ(1-42) samples that had aggregated in the presence of the DMPC:GM1 LUVs were less toxic to cultured human SH-SY5Y neuroblastoma cells than samples that had aggregated in absence of lipid vesicles (Fig. 4c), strengthening the notion that GM1 may have protective impact on the Aβ(1-42) aggregation cascade.

### Cholesterol and sphingomyelin accelerate Aβ(1-42) aggregation by different mechanisms

**Fig. 5:**
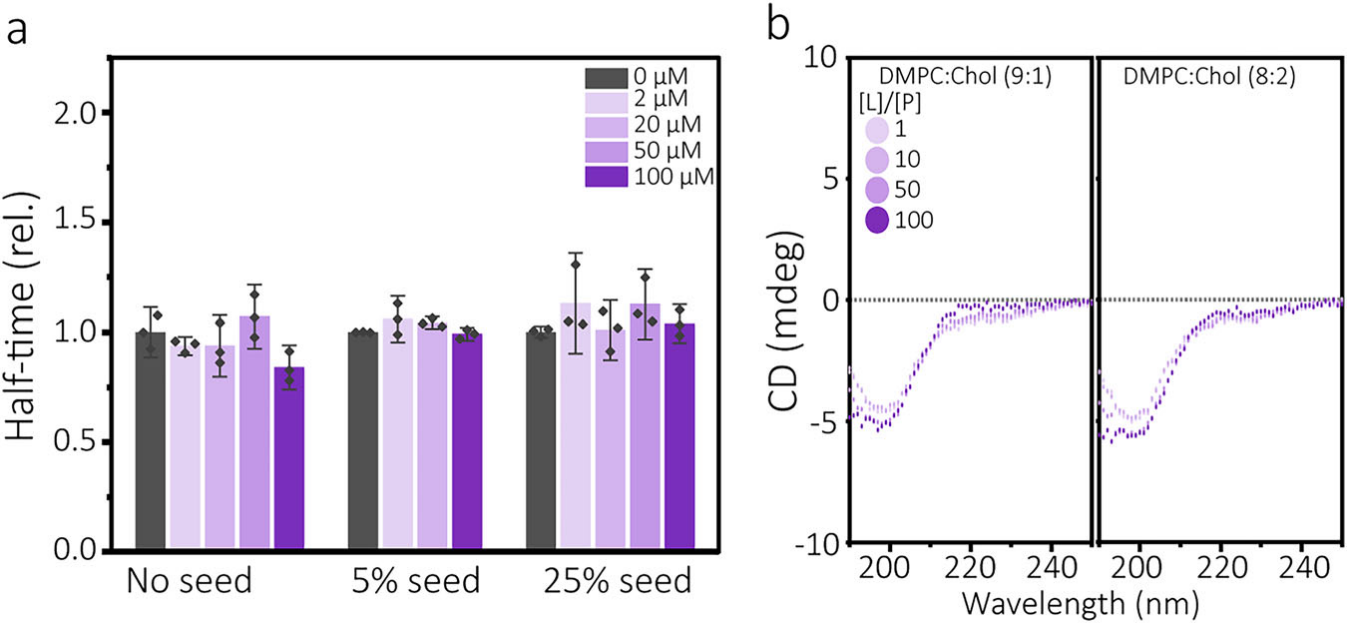
Analysis of the acceleratory Cholesterol-Aβ(1-42) interaction. **a** Change in half-times of seeded Aβ(1-42) aggregation in presence of increasing concentrations of the DMPC:Chol (8:2) LUVs. Data are reported relative to the half-times of seeded Aβ(1-42) aggregation in buffer. The corresponding kinetic curves are shown in Fig. S6b and Fig. S16c and d. Error bars represent standard deviation of the mean of three technical replicates (n = 3). **b** Circular dichroism (CD) spectra of 10 µM Aβ(1-42) monomer solutions recorded immediately upon addition of LUVs at a molar concentration ratio, [L]/[P] of1, 10, 50, or 100(DMPC:Chol (9:1) LUVs in left window or DMPC:Chol (8:2) in right window).

The kinetic analysis showed that Chol and SM, as opposed to GM1, catalysed Aβ(1-42) fibril formation when present in binary composition (DMPC:X) LUVs (Fig. 2a, Supplementary Fig. 6a, b, e and f). The results for Chol agrees well with previous reports^21,52^. Analysis and fitting of the kinetic data (Fig. 3d, Supplementary Fig. 13e–h) support the notion that Chol accelerates primary nucleation^21^. This was further confirmed by observations that DMPC:Chol LUVs lost their catalytic effect in presence of pre-formed Aβ(1-42) fibril seeds (Fig. 5a, Supplementary Fig. 16c and d). We speculate that Chol, due to its small hydroxyl head-group and consequent ability to increase the spacing and mobility in the head-group regions of lipid bilayers^53^, could facilitate binding of soluble Aβ(1-42) at the membrane interface. CD spectra recorded immediately upon addition of DMPC:Chol LUVs to solutions of monomerised Aβ(1-42) suggest the formation of β-sheet structure (Fig. 5b), supporting this idea.

SM lipids also catalysed Aβ(1-42) aggregation, but by a mechanism that resulted in an increase in the *k*_*+*_*k*_*2*_ rate constant (Fig. 3d, Supplementary Fig. 13i–l), suggesting that SM enhances secondary nucleation. We could not observe any interactions between soluble Aβ(1-42) and DMPC:SM LUVs using CD due to the high intrinsic CD of membrane-associated SM (Supplementary Fig. 17) which overlaps with and obscures the weaker peptide-associated peaks^54^. Others have, however, reported that SM lipids can enhance Aβ(1-42) oligomerisation^41^.

### Competition between delay and catalysis of Aβ(1-42) aggregation arises in LUVs with mixed lipid composition

Having established GM1 as a kinetic inhibitor, and Chol and SM as catalysts of Aβ(1-42) aggregation, we explored what happens when lipids with opposite intrinsic effects are mixed into the same lipid bilayer. Two conceptually different trends emerged. First, in DMPC:SM:GM1 LUVs, the SM-associated catalytic effect on Aβ(1-42) aggregation was entirely dominant, overruling the inhibitory effect associated with GM1 (Figs. 2a, 3d, Supplementary Fig. 6i and j). The trend was even such that the increase in Aβ(1-42) aggregation half-times were larger with DMPC:SM:GM1 LUVs compared to binary composition DMPC:SM LUVs (Fig. 2a). This suggests that GM1 potentiated SM-mediated catalysis, rather than competed with it. By contrast, with DMPC:Chol:GM1 LUVs, we observed direct competition (Figs. 2a, 3d, Supplementary Fig. 6g and h) with different outcomes depending on the total lipid concentration (and hence peptide-to-lipid ratio) in the assayed system. The Chol-mediated catalysis of Aβ(1-42) aggregation dominated at low total lipid concentrations and GM1-mediated delay dominated at high total lipid concentrations and high GM1 molar ratios (Supplementary Fig. 6g and h). This suggests that the primary nucleation mediated by membrane Chol is most effective under conditions where the peptide-to-lipid ratio, and hence local membrane-associated Aβ(1-42) concentration is high, whereas the GM1-mediated inhibition scales with available Aβ(1-42) binding sites. The switch between Chol-mediated catalysis and GM1-mediated delay occurred at total lipid concentrations between 20 and 50 μM, depending on GM1 content in the LUVs. Notably, in none of these cases did we observe that the opposing effects of two lipids simply cancelled.

### Membrane rigidity potentiates GM1-mediated delay of Aβ(1-42) aggregation and out-competes catalytic effects of other lipids

We finally explored how LUVs with quaternary (DMPC:SM:Chol:GM1) compositions affect Aβ(1-42) aggregation. SM:Chol:GM1 mixtures are known to promote the formation of lipid rafts^55^, which was also reflected by low membrane fluidity (Fig. 1b). The quaternary LUVs had strong inhibitory effects on Aβ(1-42) aggregation kinetics (Fig. 2a) and this resulted from extended lag times (Fig. 2b) and reduced primary nucleation rates (Fig. 3d), consistent with a potentiation of the inhibitory mechanism of GM1. This provides a second example of where some lipids (Chol, SM) seemingly give up their intrinsic, in this case catalytic, behaviours and instead potentiate the inhibitory effect on aggregation kinetics of another (GM1), emphasising the importance, not only of individual chemical properties of lipids, but their collective contribution to shape membrane physical properties.

**Fig. 6:**
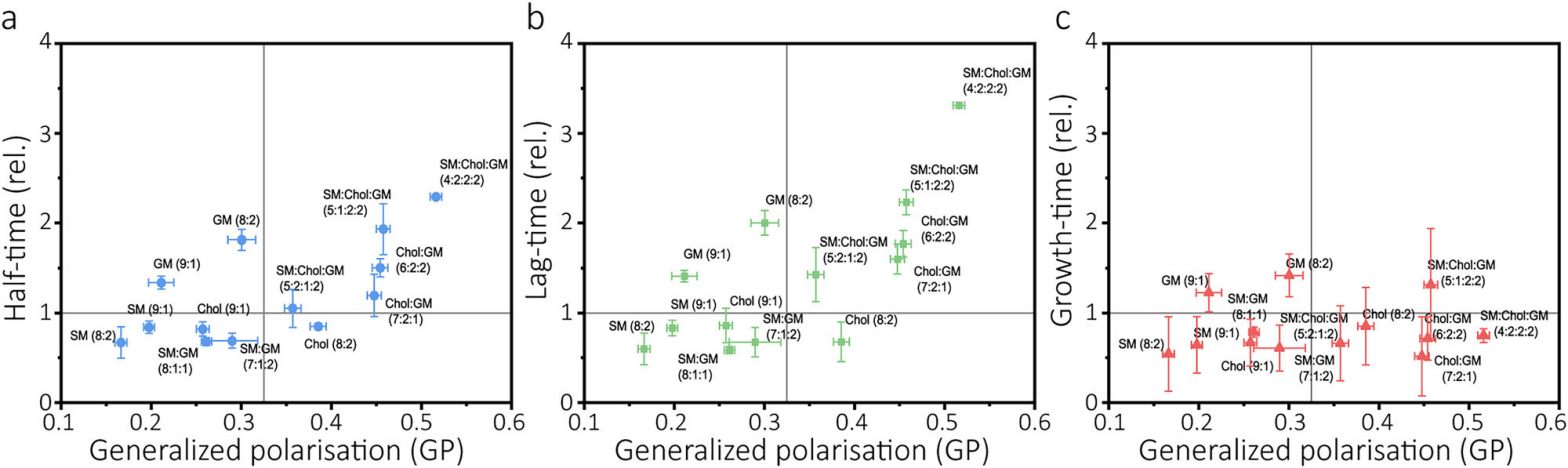
Correlations of membrane fluidity and Aβ(1-42) aggregation parameters. Correlations of **a** half-times, **b** lag-times, and **c** growth-times of Aβ(1-42) aggregation with laurdan generalised polarisations (GP), based on data from Figs. 1b and 2a–c.The solid grey line at y = 1 represents the kinetic parameters for Aβ(1-42) in buffer and the corresponding line at x = 0.325 represents the laurdan GP regime in which Chol-containing membranes transition from L_d_ to L_o_/L_d_ phase transition^21^. Error bars represent standard deviations as reported in Figs. 1b and 2a–c.

We examined the correlation between membrane rigidity (laurdan GP) and the different Aβ(1-42) aggregation reaction parameters presented in Fig. 2. We found that reaction half-times (Fig. 6a) and lag-times (Fig. 6b) scale with membrane rigidity, whereas growth-times are invariant to fluidity change (Fig. 6c), consistent with their invariance to lipid composition (Fig. 2d). This is consistent with that membrane rigidity has been reported to facilitate GM1 clustering, which in turn may potentiate Aβ(1-42) binding. Generally, this analysis shows that catalytic lipid effects only persist in low complexity LUVs, and that it is the enhanced membrane rigidity that potentiates GM1’s effect on the delay of Aβ(1-42) aggregation. However, two groups of LUVs did not confine to this definition; DMPC:Chol (8:2) LUVs have low fluidity but retain their catalytic effect in absence of GM1 and DMPC:GM1 are obligate inhibitors of Aβ(1-42) aggregation kinetics even when membrane fluidity is high. The latter suggests that Aβ(1-42) binding may induce local GM1 clusters which are not large enough to be detected by the laurdan assay.

## Discussion

Disruption of brain lipid homoeostasis is increasingly linked to the pathology of neurodegenerative diseases, including Alzheimer’s disease^9,10,56^. Results from both cell and animal studies have shown that alterations to lipid composition can directly alter pathogenic protein aggregation, for example levels of toxic amyloid species^57,58^, and membrane lipids are commonly co-deposited with amyloid fibrils in plaques and protein inclusions^59^. Deciphering this intersection is important to understand mechanisms of protein aggregation in disease.

In this study, we have explored how model membranes with systematic variations in lipid composition affect the kinetics and mechanisms of Aβ(1-42) amyloid formation in vitro. The underlying motivation was that many brain-relevant lipids^60–62^, including phospholipids^23^, Chol^21^, glycolipids^41,43^, and SM^41,63^, have been reported to individually either catalyse or inhibit the oligomerisation and fibril formation of Aβ(1-42), but rationalising the net effect of these lipids in a mixed, and hence more biologically relevant, bilayer has remained a challenge.

Our kinetic study and mechanistic analysis show that GM1 delayed and Chol catalysed Aβ(1-42) aggregation, both at the level of primary nucleation. SM, on the other hand, affected aggregation by a mechanism that is consistent with the catalysis of secondary nucleation. We have previously shown that extracellular vesicles (EVs) can slow down Aβ(1-42) fibril elongation^24^. Altogether, this highlights a significant diversity and suggests that biological membranes contain components that are capable of regulating every key step in the Aβ(1-42) reaction cascade. This suggests that biological membranes have an inherent ability to exert versatile control over amyloid formation.

Whereas our mapping of the catalytic effect of Chol primarily confirms and extends previous data^21^, the GM1 mechanism deserves further discussion as the literature is highly ambiguous, with reports of both aggregation catalytic^42,51,64–66^ and inhibitory^41,67^ effects. This discrepancy may result from GM1’s tendency to partition into micelles and other non-bilayer assemblies, which may, furthermore unintendedly, co-exist with vesicles if the GM1 concentration in the assayed system is too high^64,68^. However, other studies that have directly focused on non-membranous GM1 have reported catalysis of Aβ aggregation^64^ as well as inhibition^67^. Our study focuses on membrane-bound GM1 and we report primary nucleation inhibition across a range of membrane conditions, as well as a significant enhancement of the effect if the model membranes have low fluidity as is associated with lipid raft formation. This is consistent with a wide body of literature on the in vitro and in vivo formation of Aβ-GM1 complexes and clusters^66,69–72^ and our data further supports the notion that GM1 promotes Aβ oligomerisation, but aligns with the view that these are off-pathways amyloid species^41^ with low toxicity^67^. Research have shown a gradual decline of GM1 levels in the human brain during healthy ageing as well as in AD^18,32,34,36,73^. It has been suggested that non-membranous GM1 structures in the brain may accumulate from debris of dying neurons during the course of disease, and some literature suggests a redistribution of GM1 within neuronal membranes to regions of raft-like nature^69,71,74^. A possible rationalisation of our data in the context of disease is that GM1 normally acts as a sink for soluble Aβ, preventing its pathological aggregation. However, this protection may diminish by ageing and even reverse upon neuronal damage in AD, with raft-association of GM1 in AD brains being a possible counter-acting mechanism.

Although individual lipids may have significant effects on protein aggregation, especially if they interact directly with the protein in question (as for GM1), membrane lipids exist in a complex and compositionally diverse two-dimensional matrix in biology. A key purpose of this study has been to provide mechanistic insight into how Aβ(1-42) aggregation proceeds in presence of membranes with both catalytic and inhibitory components.

By systematic variation in the mixing of GM1, Chol and SM lipids into complex model membranes, we show that the aggregation modulatory effects of mixed lipid membranes are not simply the sum of the effects of their individual lipid constituents, meaning that opposing effects rarely cancel. Instead, we observe several examples of where one lipid ‘wins’ and where its effect can even be potentiated by presence of a lipid with intrinsic opposite aggregation modulatory function. This contrasts a suggestion, based on observations using mixed phospholipid membranes, that lipid complexity would induce a resilience in the membrane to its modulation of Aβ(1-42) aggregation^25^ and is more in line with the fact that true biological membranes, such as EVs, have distinct aggregation modulatory effects on Aβ(1-42)^24^, as well as on other proteins^35^. Our work furthermore highlights two interesting principles.

First, when GM1 was mixed with either Chol or SM into membranes that were fluid and incapable of forming lipid rafts, we observed either direct competition (between GM1 and Chol) or situations where the aggregation-modulatory effect of one component (SM) dominated the outcome of Aβ(1-42) self-assembly. We reason that the direct competition between GM1 and Chol arises because both lipids intrinsically catalyse primary nucleation, e.g., the first step in the protein aggregation cascade. SM, on the other hand acts on secondary processes which are responsible for the rapid and self-perpetuating nature of Aβ(1-42) aggregation^48^. Therefore, once a few primary nuclei have formed, this catalysis may proceed undisturbed by the GM1 lipid. In addition, SM was observed to increase membrane fluidity, in DMPC LUVs, presumably because of the significant acyl chain unsaturation in the brain-derived extract used, but potentially also due acyl chain length mismatch as a significant proportion of the SM lipids were > 20 carbons in length. This may in fact diminish the formation of GM1:Aβ(1-42) complexes, which are accentuated by GM1-clusering^38^. These two examples highlight that the outcome of mixing aggregation-modulatory lipids with opposite effects is not only dependent on their relative modulatory strengths, but also on how the lipids mechanistically interfere with protein aggregation.

Second, when GM1, Chol, and SM were mixed into bilayers with increased rigidity (e.g., resembling lipid-raft conditions^55,56,69^), we observed that the collective contribution of lipids to shape a membrane’s physical properties, even within relatively simplistic membrane model systems, can profoundly alter protein aggregation rates. Specifically, it appears that Chol, when engaged in lipid-raft formation, gives up its intrinsic aggregation-catalytic role in favour of promoting GM1-clustering. It is possible that this behaviour has a relatively simple explanation in that Chol’s intrinsic aggregation-catalytic effect is unlikely to be due to specific chemical interactions with Aβ(1-42), but rather result from the increased spacing that Chol introduces in the head-group region of the bilayer, hence enabling proteins to form hydrophobic interactions at the membrane interface^75^. Lipid rafts, being characterised by tight lipid packing^55^ would likely counteract this function. From a pathological perspective, this is consistent with results of Sponne et al. showing that Chol depletion increases Aβ toxicity^64^ despite its intrinsic ability to drive Aβ(1-42) aggregation.

In conclusion, this study rationalises mechanistically the role of three lipids with high AD relevance as modulators of Aβ(1-42) aggregation and explore how they act in consort. Our work shows that lipid membranes can have potent and diverse effects on all assembly steps that determine the rate of Aβ(1-42) fibril formation. We furthermore demonstrate that lipid-mediated protein aggregation is ultimately not controlled by chemical properties of individual lipids and highlights the significance of lipid context, such as the fluidity of the membrane and its lateral organisation. This suggests that cells, by virtue of the complexity of their membranes, can exert fine-tuned control over the solubility and aggregation of amyloidogenic proteins and that disturbance in lipid homoeostasis during ageing or disease, may offset this control, and hence drive pathological aggregate formation.

## Methods

### Aβ(1-42) expression and purification

Recombinant Aβ(1-42) was expressed in E.coli as a fusion protein with the solubility tag NT*^76,77^, cleaved with tobacco etch virus (TEV) protease (produced as described by Tropea at al.^78^) and purified as previously described^79^. The purified Aβ(1-42) was stored in freeze dried aliquots at −20 °C until further use. Immediately prior to each aggregation kinetic and circular dichroism (CD) experiment, lyophilised Aβ(1-42) was dissolved in 6 M guanidium hydrochloride on ice for 20 min, and thereafter monomerized by size exclusion chromatography (SEC) on a Superdex 75 10/300 column (GE Healthcare) in 20 mM sodium phosphate buffer, pH 8.0. The monomeric Aβ(1-42) was eluted as a single peak at approximately 14 mL, as previously reported by us^79^ and others^48^. The concentration in the collected monomer fraction was determined by integration of the monomer peak in the chromatogram (Supplementary Fig. 4) (ε_280nm_ = 1280 M^-1^ cm^-1^).

### Preparation of large unilamellar vesicles (LUVs)

LUVs were prepared by mixing chloroform-dissolved lipids at desired molar ratios followed by formation of a dry lipid film by rotary evaporation and drying under vacuum (>4 h). The lipid film was hydrated in 20 mM sodium phosphate buffer, pH 8.0 by vortexing (10 min). The resulting solution was extruded through a 100 nm polycarbonate filter 21 times. LUVs were stored at 4 °C and used within 2 weeks. The following lipids, purchased from Avanti Polar Lipids, were used in this study: 1,2-dimyristoyl-*sn*-glycero-3-phosphocholine (DMPC) (cat. no. 850345), 1,2-dioleoyl-*sn*-glycero-3-phosphocholine (product no. 850375) cholesterol (plant, cat. no. 700100), monosialotetrahexosylganglioside GM1 (Ovine brain, cat. no. 860065) with a fatty acid composition of ~80% 18:0, ~19% 20:0, and ~1% 18:1^68^, and sphingomyelin (Porcine, brain, cat. no. 860062) with a fatty acid composition, given by the manufacturer, of 2% 16:0, 50% 18:0, 5% 20:0, 7% 22:0, 5% 24:0, 21% 24:1, and 10% others.

### Thioflavin-T (ThT) monitored aggregation kinetics

Samples containing 2 µM of freshly monomerized Aβ(1-42) (purified as described above) in 20 µM sodium phosphate buffer with 5 µM thioflavin-T (ThT, Sigma Aldrich), and 0–100 µM of LUVs (lipid equivalents) were prepared on ice and then rapidly distributed, in 70 μL triplicates, to the wells of Corning #3881 96-well black half-area microtiter plates with transparent bottom. The plates were sealed with adhesive film (BIO-RAD, Hercules, CA, US) and the ThT emission was read as function of time in a FluoStar OPTIMA fluorescence plate-reader (BMG Labtech) operated at 37 °C, without shaking and using bottom optics, and 440 ± 10 nm and 490 ± 10 nm bandpass filters for excitation and emission. For seeded aggregation, fibrils from a previous experiment were used. All kinetic experiments were performed in technical triplicate and repeated on at least three separate occasions.

### Analysis and modelling of kinetic data

Half-times, lag-times and growth-times were extracted from normalised ThT kinetic curves. Lag-times were defined as the time taken to reach 10% of the maximum ThT intensity, and growth-times as the time taken for the normalised ThT fluorescence intensity to increase from 0.1 to 0.9. The kinetic data was fitted using a secondary nucleation dominated aggregation model with saturation^50^ in AmyloFit^50^. The model operates with compounded rate constants for elongation and primary nucleation (k_+_k_n_), and elongation with secondary nucleation (k_+_k_2_) and a Michelis-Menten type constant (K_M_) to describe saturation, which was determined by fitting the model to kinetic data for Aβ(1-42) aggregation in buffer. To determine the relative importance of primary and secondary processes, we performed two fittings per data set setting either k_+_k_n_ or k_+_k_2_ as a global constant. The goodness of fit (mean residual error) was used to determine the dominant aggregation-modulatory mechanism in presence of different LUVs.

### Atomic force microscopy (AFM)

Fibril samples were deposited onto freshly cleaved mica that had been functionalized with 0.5% (v/v) (3-aminopropyl) triethoxysilane (Sigma) in Milli-Q water for 1 min, rinsed with milli-Q water and dried with nitrogen gas. 10 µL of sample was left to settle for 10 min, followed by 5X rinsing with Milli-Q water and drying of the samples with nitrogen gas. AFM images were acquired using an NTEGRA Prima (NT-MDT) setup with a gold coated single crystal silicon cantilever (NT-MDT). 256 × 256 pixel images were captured of 5 × 5 µm areas using a 1.01 Hz scan rate. A minimum of 200 Aβ(1-42) fibrils were measured for fibril length and height for Aβ(1-42) fibrils formed in absence (buffer) or presence of the different LUVs. Between 6 and 22 images were acquired per sample (exact numbers of images taken per sample can be found in Supplementary Information, Supplementary Figs. 8–11). Images were processed using in Gwyddion by planar subtraction, polynomial background subtraction and correction for linear aberrations as previously described^80^.

### Circular dichroism (CD) spectroscopy

10 µM of freshly monomerised solutions of Aβ(1-42) were mixed with LUVs solutions and incubated on ice for 6 min. CD spectra were then recorded on a Chirascan spectropolarimeter (Applied Photophysics) in a 1 mm quartz cuvette with a scan speed of 1 nm/min. 4 spectra were recorded and averaged. All spectra were corrected for background contributions by subtracting blanks (buffer with LUVs).

### Cytotoxicity

SH-SY5Y human neuroblastoma cells were maintained in 1:1 medium of MEM + GlutaMAX and F-12 Nut Mix (Gibco, USA), supplemented with 10% FBS and 1% non-essential amino acids (Gibco, USA) and sub-cultured every 4 days. The cell line identity was confirmed by CLA testing, and all cultures were confirmed mycoplasma free by PCR-based tests (Eurofins genomics). Cells were plated at a density of 20,000 cells per well in 96 well plates, one day prior to cytotoxicity experiments. The cells were then exposed for 24 h to 1 µM of Aβ(1-42) aggregated in absence or presence of LUVs in serum-free medium. The Alamar blue assay was used to assess the cytotoxicity of samples which were collected at the end-point of the aggregation kinetic experiments and added to the cells without any further purification. Alamar blue measures the reducing power of living cells via the conversion of resazurin into resorufin. The cells were washed 3× in DPBS, and incubated with Alamar blue reagent (manufacturer) at a dilution of 1:10. The fluorescence of resorufin was then detected using a FLUOstar OPTIMA plate reader and 544/10 nm and 590/10 nm excitation and emission bandpass filters. The data were corrected for background contributions by subtracting cell medium with added substances (Aβ(1-42) and different concentration of LUVs) as blanks. The metabolic activity in each cell sample was taken as the average of three technical replicates (n = 3). All experiments were performed in biological triplicate (N = 3).

### Laurdan fluorescence

Laurdan stock solutions (10 mM) were prepared in dimethylformamide and stored at −20 °C until further use. LUVs (1 mM) and laurdan (10 µM) were incubated at 45 °C for 30 min to equilibrate the dye. Each samples was thereafter diluted in 20 mM phosphate buffer (pH = 8.0) and dispensed into 96-well black half-area microtiterplates with transparent bottom ((Corning #3881). Laurdan fluorescence was collected across different temperatures using a BMG Clariostar Plus plate reader with a 350/15 nm excitation filter and 460/14 nm and 500/15 nm emission filters. The laurdan generalised polarisation values were calculated as GP = (I_460_ - I_500_)/(I_460_ + I_500_).

### Statistics

All Aβ(1-42) aggregation kinetics experiments were performed as at least three independent repeats (N = 3), each in technical replicate (n = 3). Data in the manuscript show one representative data set (N = 1), with three technical replicates (n = 3) and are reported as mean and with error bars representing standard deviation. Pearson’s correlation coefficients were calculated using OriginPro Software to assess the linear relationship between Aβ(1-42) lag-times and half-times in presence of LUVs, as described in the Results section. Cytotoxicity is reported as mean ± standard deviation of three biological replicates (N = 3), performed in triplicate (n = 3).

### Reporting summary

Further information on research design is available in the Nature Portfolio Reporting Summary linked to this article.

## Supplementary information

Supporting tables and figures

Reporting Summary

## Data Availability

Data that support the findings of this study have been deposited on figshare.com, 10.6084/m9.figshare.30674660.
