# Supplementary material for: Ganglioside GM1 slows down Aβ(1-42) aggregation by a primary nucleation inhibitory mechanism that is modulated by sphingomyelin and cholesterol
Source: Commun Chem. 2025 Dec 13;9:39. doi: 10.1038/s42004-025-01846-y (PMC12824409; doi:10.1038/s42004-025-01846-y)
Supplement: Supplementary file 1 — Supporting tables and figures [file 42004_2025_1846_MOESM1_ESM.pdf]

Supplementary information to

Ganglioside GM1 slows down Aβ(1-42) aggregation by a primary nucleation inhibitory mechanism that is modulated by sphingomyelin and cholesterol

Nima Sasanian, Vesa Halipi, Mikaela Sjögren, Johannes Bengtsson, David Bernson, Elin K. Esbjörner*

Division of Chemical Biology, Department of Life Science, Chalmers University of Technology, Kemivägen 10, 412 96 Gothenburg, Sweden

*Corresponding author: Elin K. Esbjörner, eline@chalmers.se

Supplementary Table 1. Lipid vesicle compositions. The table shows the compositions of the lipid vesicles used in the study.

Complexity	Lipid Components	Denotation (Mole fraction)	SM	Chol	GM1
Single component	DMPC	pure	-	-	-
	DOPC	pure	-	-	-
Binary	DMPC:GM1	9:1<br>8:2	-	-	10%<br>20%
	DOPC:GM1	9:1<br>8:2	-	-	10%<br>20%
	DMPC:Chol	9:1<br>8:2	-	10%<br>20%	-
	DMPC:SM	9:1<br>8:2	10%<br>20%	-	-
Ternary	DMPC:SM:GM1	8:1:1<br>7:1:2	10%	-	10%<br>20%
	DMPC:Chol:GM1	7:2:1<br>6:2:2	20%	-	10%<br>20%
Quaternary	DMPC:SM:Chol:GM1	6:1:2:1<br>5:1:2:2	10%	20%	10%<br>20%
		6:2:1:1<br>5:2:1:2	20%	10%	10%<br>20%
		5:2:2:1<br>4:2:2:2	20%<br>20%	20%<br>20%	10%<br>20%

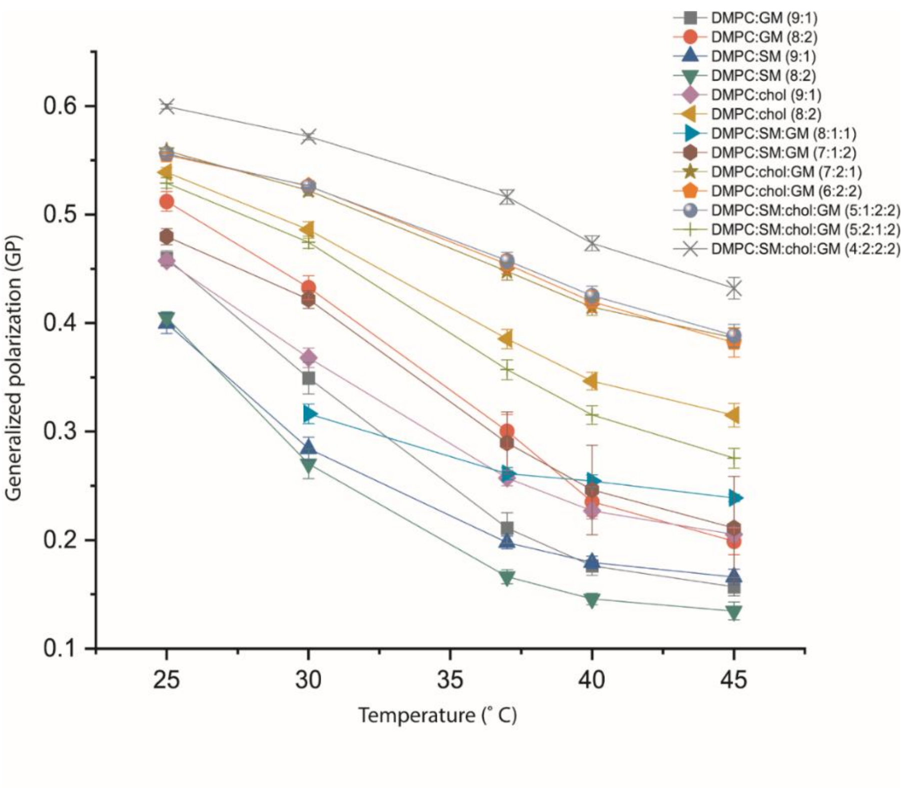

Supplementary Figure 1. Membrane fluidity of different LUVs. Generalized polarization (GP) of laurdan recorded at different temperatures in presence of different LUV types containing DMPC, GM1, Chol, and SM showing differences in their membrane fluidity.

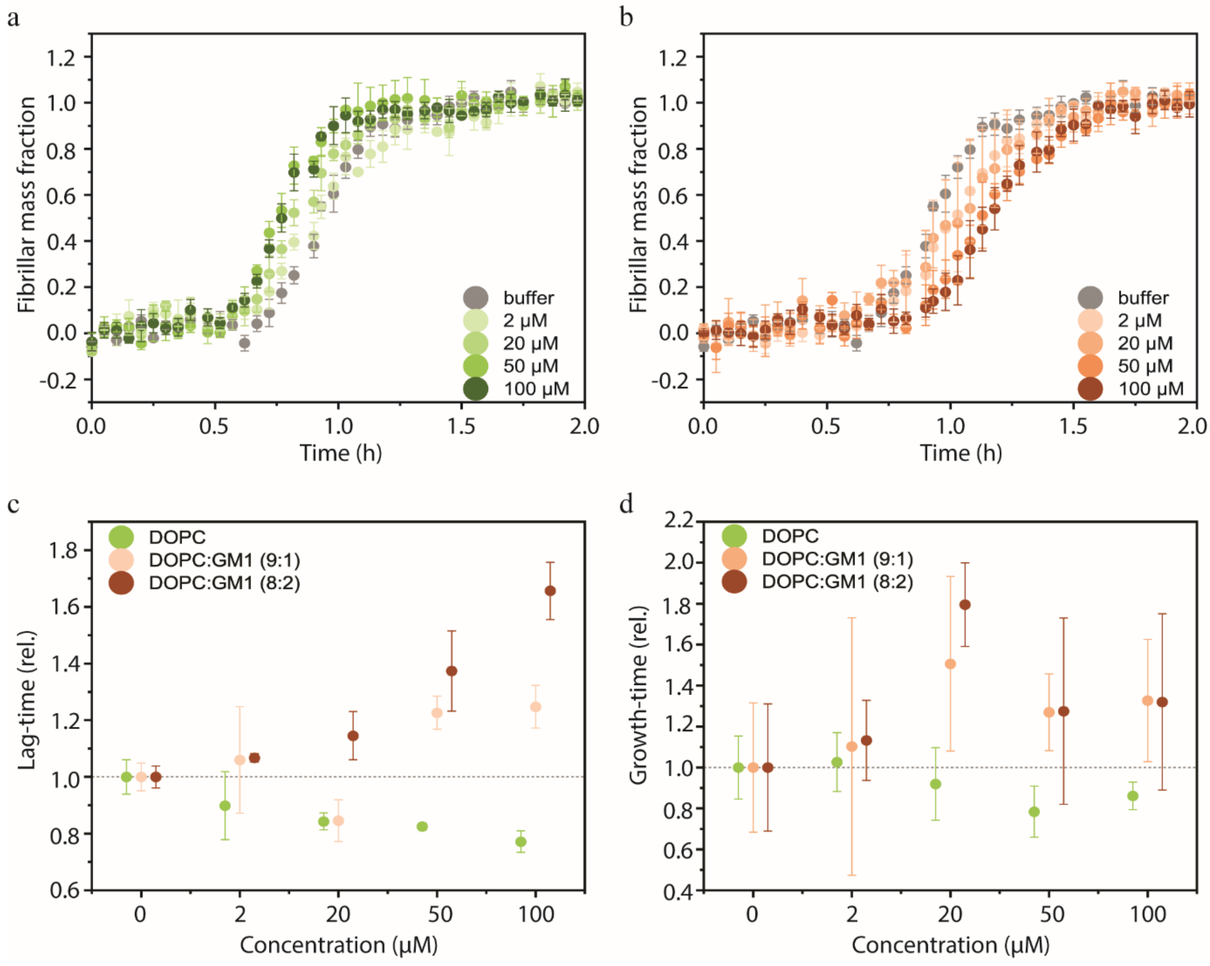

Supplementary Figure 2. Effect of DOPC:GM1 LUVs on Aβ(1-42) aggregation kinetics. Aggregation kinetics of Aβ(1-42) absence (buffer) or presence of LUVs containing DOPC and GM1. The Aβ(1-42) concentration was 2 μM and the LUV concentration was 2, 20, 50, or 100 μM (lipid equivalents). **a** Normalized kinetic curves showing the effect of DOPC LUVs **b** Normalized kinetic curves showing the effect of DOPC:GM1 (9:1) LUVs. **c** Aggregation lag-times and (**d**) growth-times across the assays concentrations of DOPC, DOPC:GM1 (9:1) and DOPC:GM1 (8:2). The data in c and d were normalized against respectively the lag-time (c) and growth-time (d) of Aβ(1-42) in buffer. The error bars represent standard deviation of the mean of three replicates (n=3).

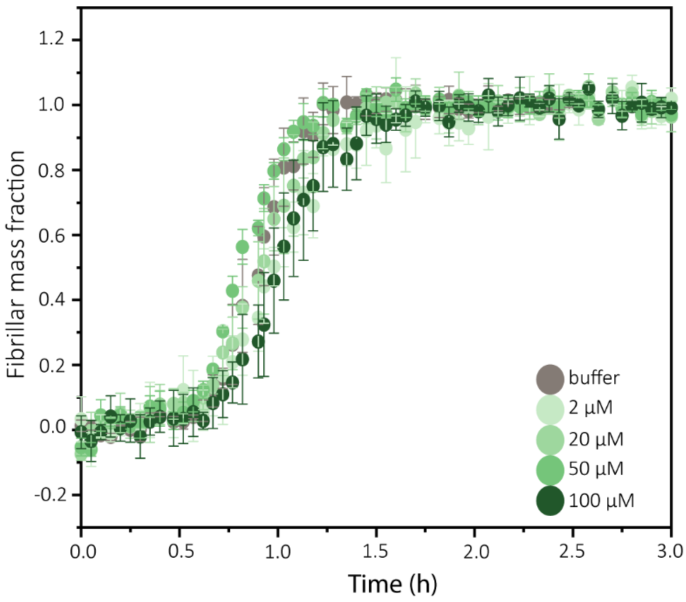

Supplementary Figure 3. Aβ(1-42) aggregation kinetics in presence of DMPC LUVs. Normalized aggregation kinetics of 2µM Aβ(1-42) in absence (buffer) or presence of 2-100 µM DMPC LUVs. The error bars represent standard deviation of the mean of three technical replicates (n=3).

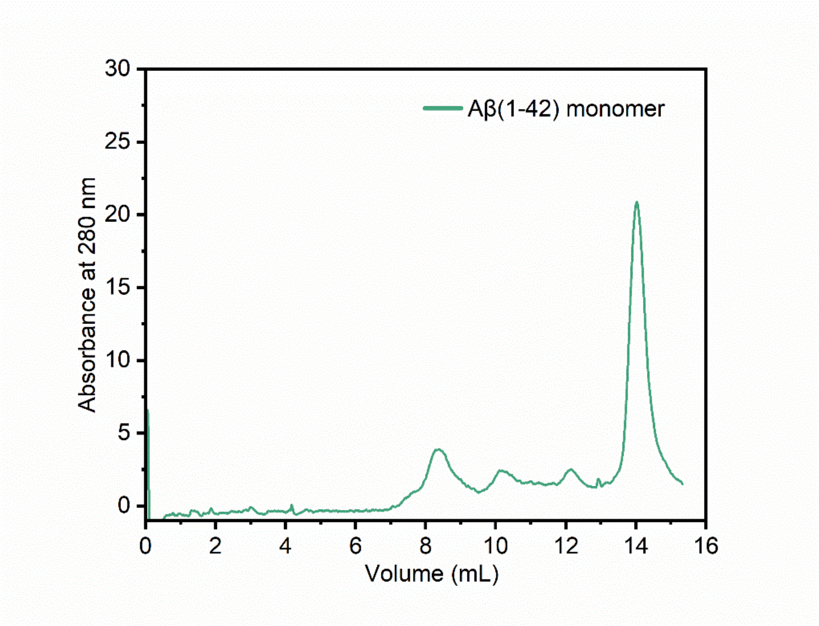

Supplementary Figure 4. Aβ(1-42) monomers purified with size-exclusion chromatography (SEC). The SEC chromatogram shows monomeric Aβ(1-42) eluted as one single peak at 14 mL from a Superdex 75 10/300 column as described in "Aβ(1-42) expression and purification" in the Methods section.

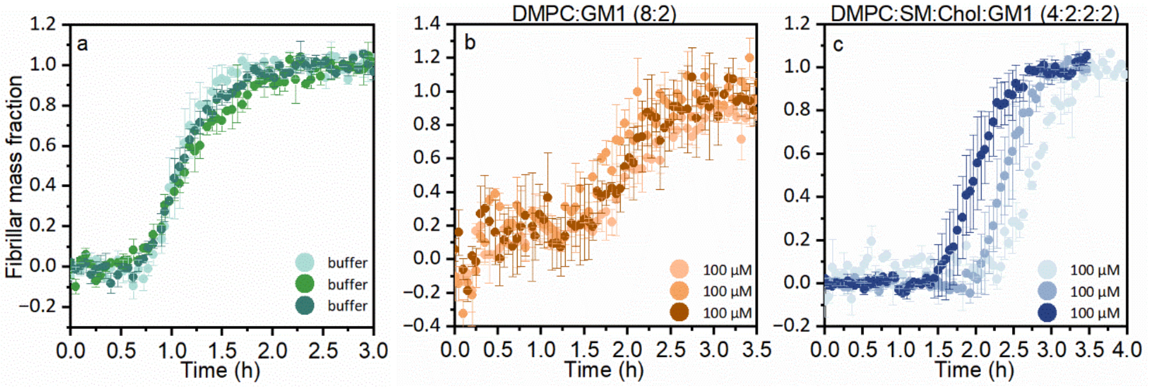

Supplementary Figure 5. Variability of Aβ(1-42) aggregation kinetics between independent repeats. Comparison of kinetic curves from three independent experiments (N=3, represented by different shades of green, orange, or blue) of the aggregation of 2 μM size-exclusion chromatography (SEC) monomerized Aβ(1-42) in absence (buffer) (**a**) or presence of DMPC:GM1 8:2 (**b**) or DMPC:SM:Chol:GM1 4:2:2:2 LUVs (**c**). Each kinetic curve represents the mean ± standard deviation of three technical replicates (n=3). See the Methods section in the main text for experimental detail.

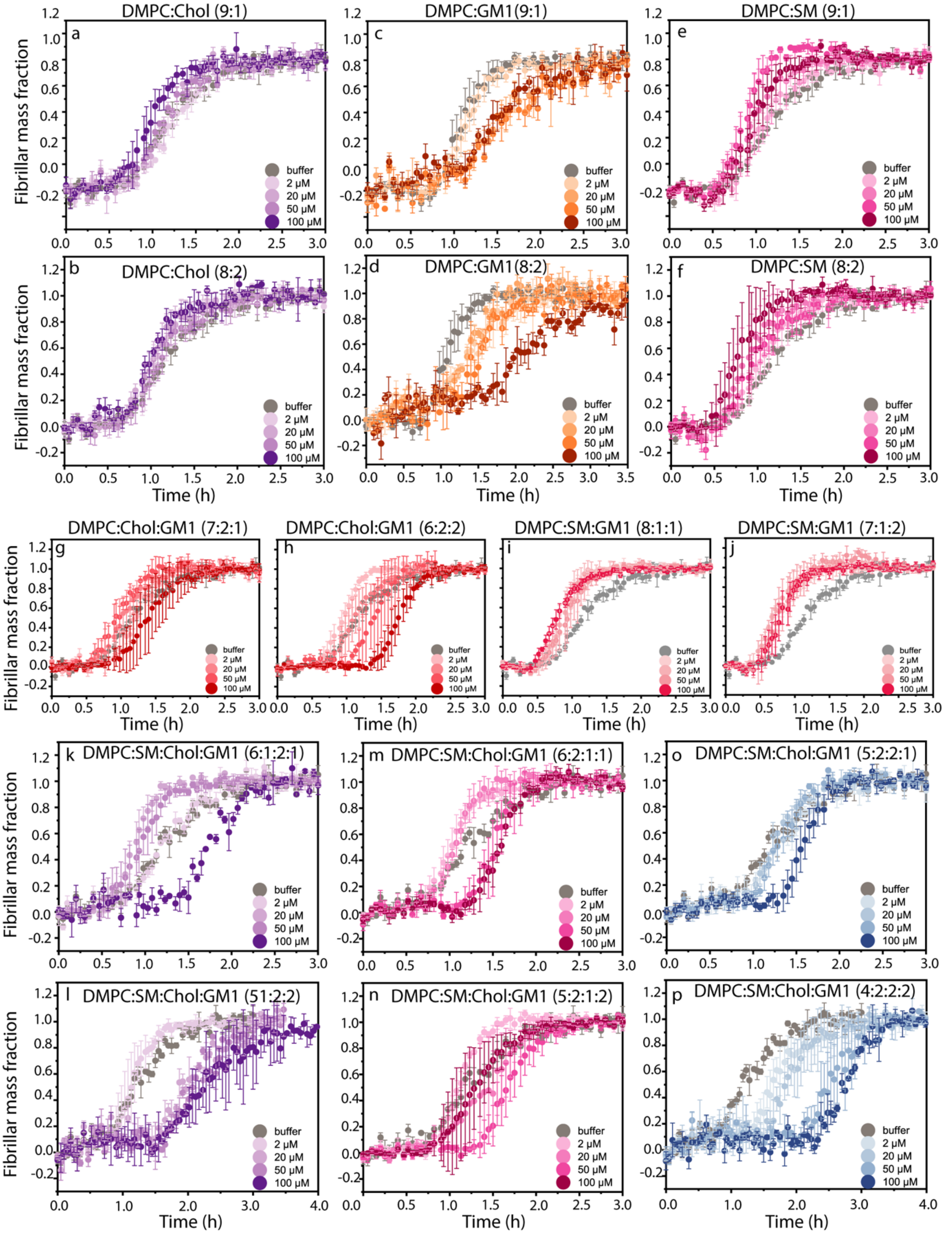

Supplementary Figure 6. Effect of different LUVs on Aβ(1-42) aggregation kinetics. Aggregation kinetics of 2 μM Aβ(1-42) in absence (buffer) or presence of 2-100 μM (lipid equivalents) of (**a-f**) binary LUVs, (**g-j**) ternary LUVs, and (**k-p**) quaternary LUVs, as denoted in the headings. The numbers in parenthesis represents molar ratios of the different lipid constituents. The aggregation kinetics were monitored by ThT fluorescence at 37°C and is represented as normalized values (fibrillar mass fraction). All kinetic experiments were repeated at least three times (N=3), the data shown represent one of these

data sets. The error bars represent the standard deviation of the mean in of three technical replicates (n=3).

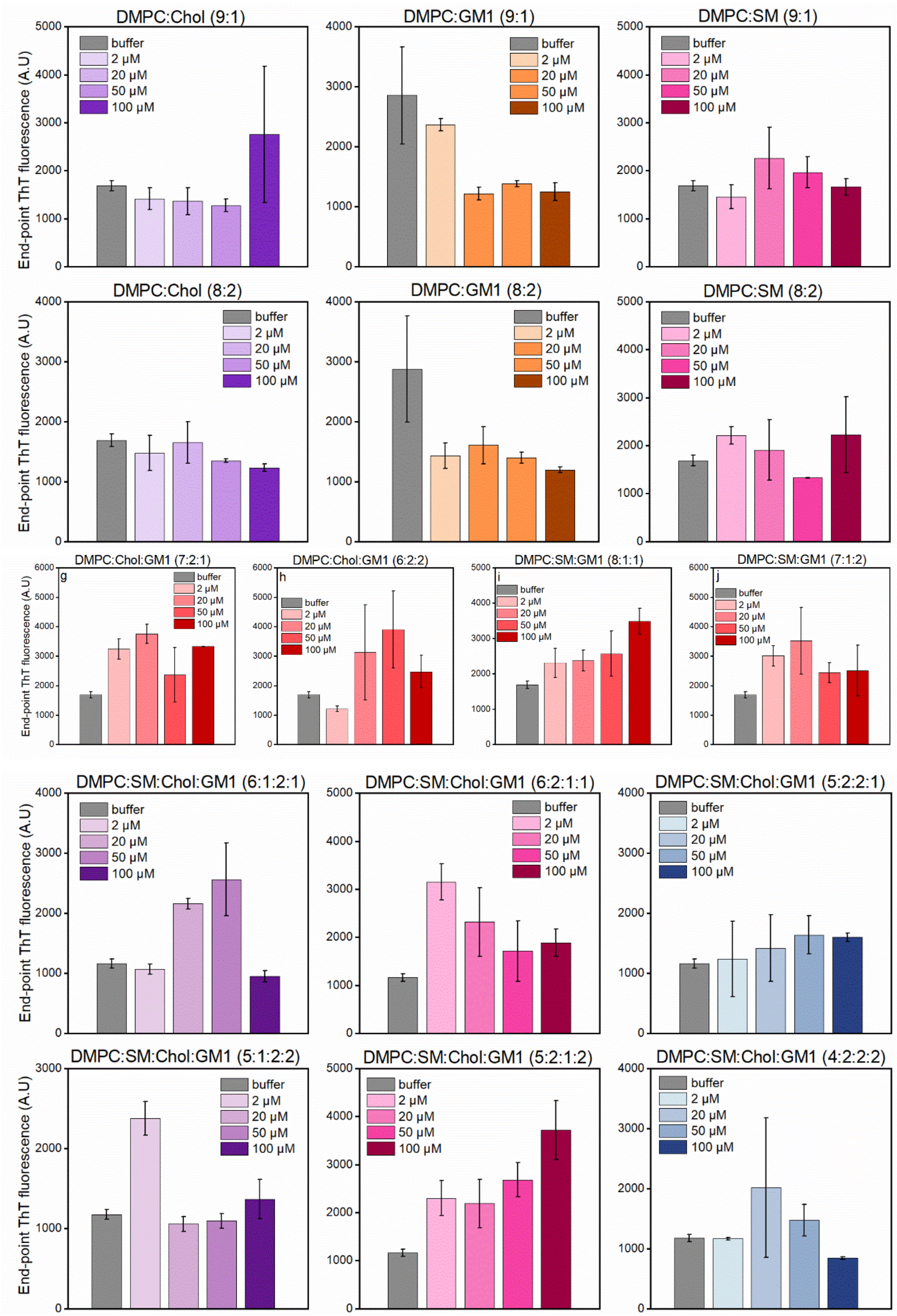

Supplementary Figure 7. End-point ThT fluorescence of Aβ(1-42) aggregation kinetics in presence of different LUVs. The end-point ThT values were extracted from the final time-point of the respective kinetic curves in Supplementary Figure 6. The bar graphs represent mean ± standard deviation (n=3).

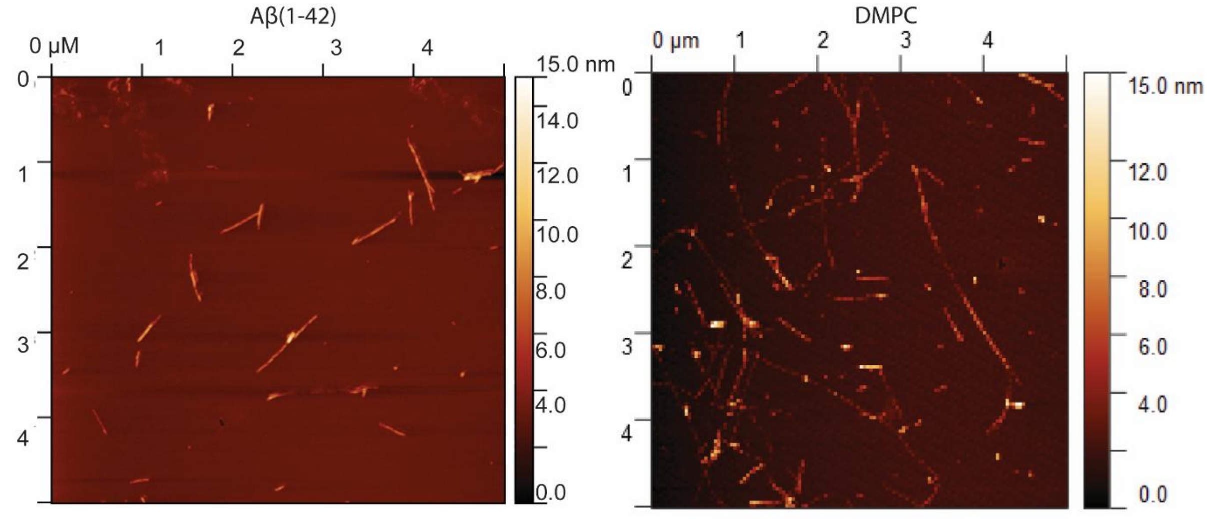

Supplementary Figure 8. Atomic force microscopy (AFM) images of Aβ(1-42) fibrils. Representative AFM images of aggregation kinetic end-point samples of Aβ(1-42) aggregated in absence (buffer) or presence of DMPC LUVs, underlying the data analysis shown in Supplementary Figure 12. .A total of 8 images were acquired for Aβ(1-42) in buffer and 6 images for Aβ(1-42) DMPC LUVs.

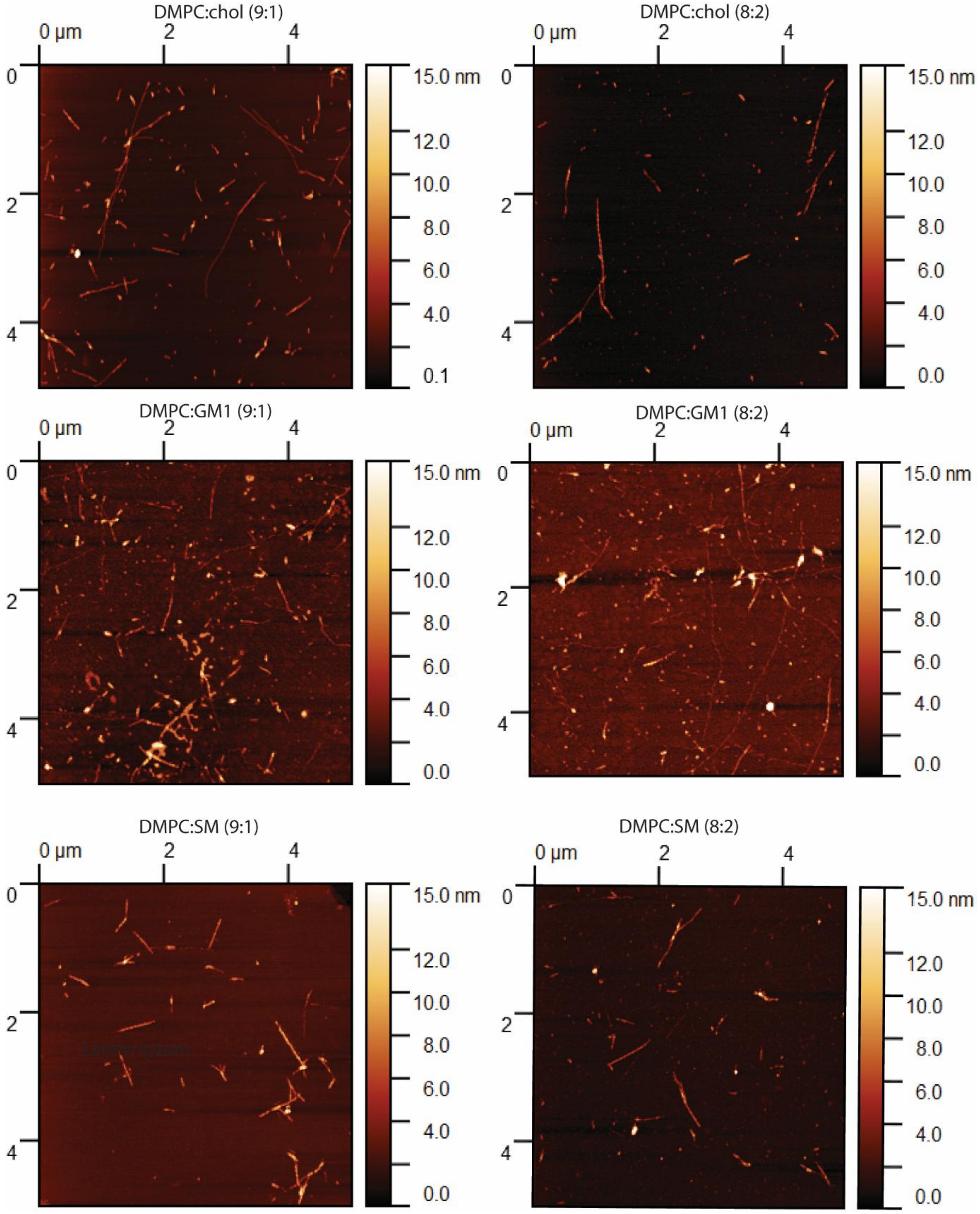

Supplementary Figure 9. AFM images of Aβ(1-42) fibrils formed in presence of binary LUVs. Representative AFM images of end-point Aβ(1-42) fibril samples formed in presence of binary LUV systems, underlying the data analysis shown in Supplementary Figure 12. A total of 7 images were acquired for DMPC:Chol (9:1) and (8:2); 13 and 15 images for DMPC:SM (9:1) and DMPC:SM (8:2), respectively; and 22 images for both DMPC:GM1 (9:1) and DMPC:GM1 (8:2).

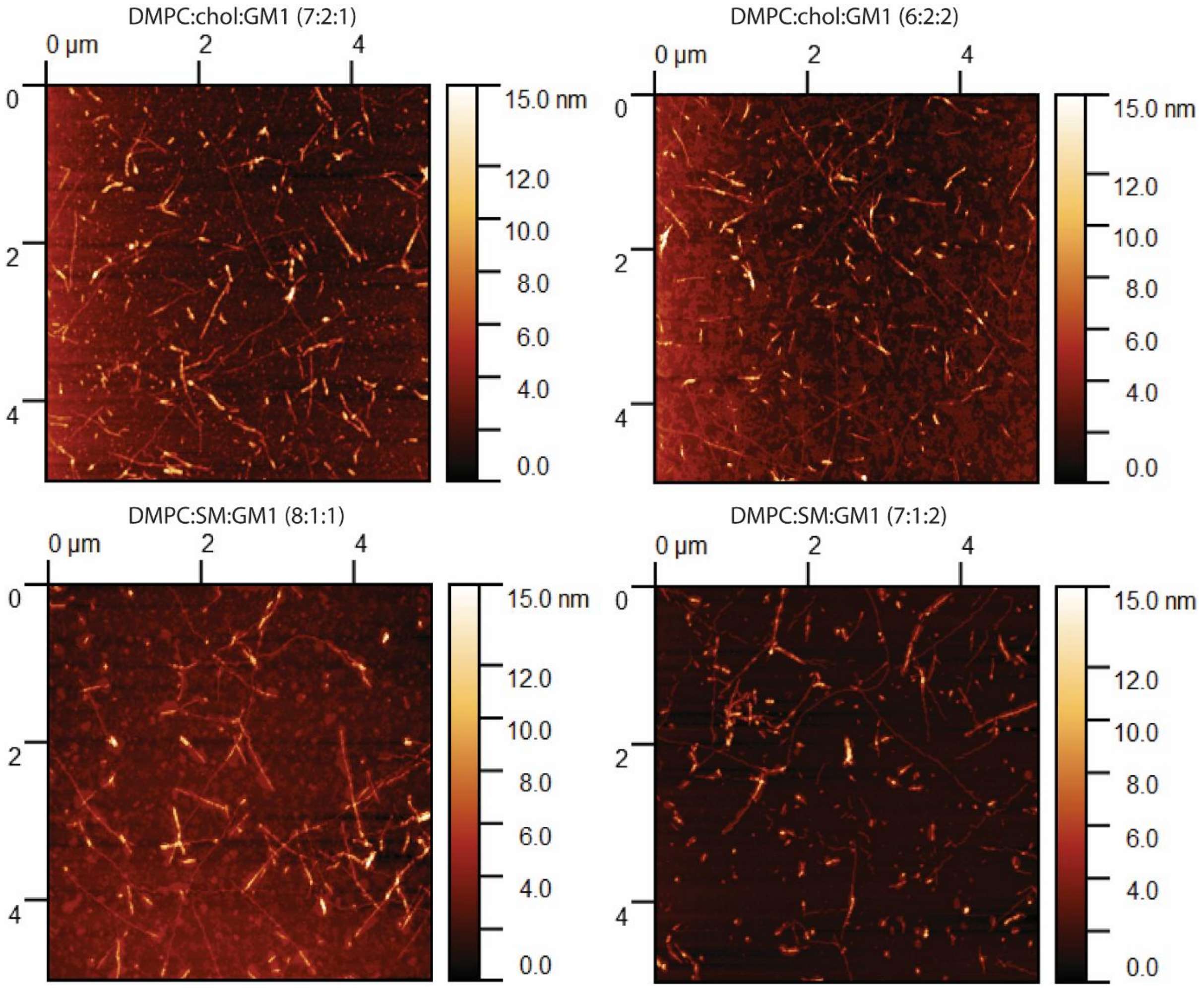

Supplementary Figure 10. AFM images of Aβ(1-42) fibrils formed in presence of ternary LUVs. Representative AFM images of end-point Aβ(1-42) fibril samples formed in presence of ternary LUV systems, underlying the data analysis shown in Supplementary Figure 12. A total of 9 images were acquired for DPMC:Chol:GM1 (7:2:1), 11 images for both DMPC:Chol:GM1 (6:2:2) and DMPC:SM:GM1 (8:1:1), and 14 images for DMPC:SM:GM1 (7:1:2).

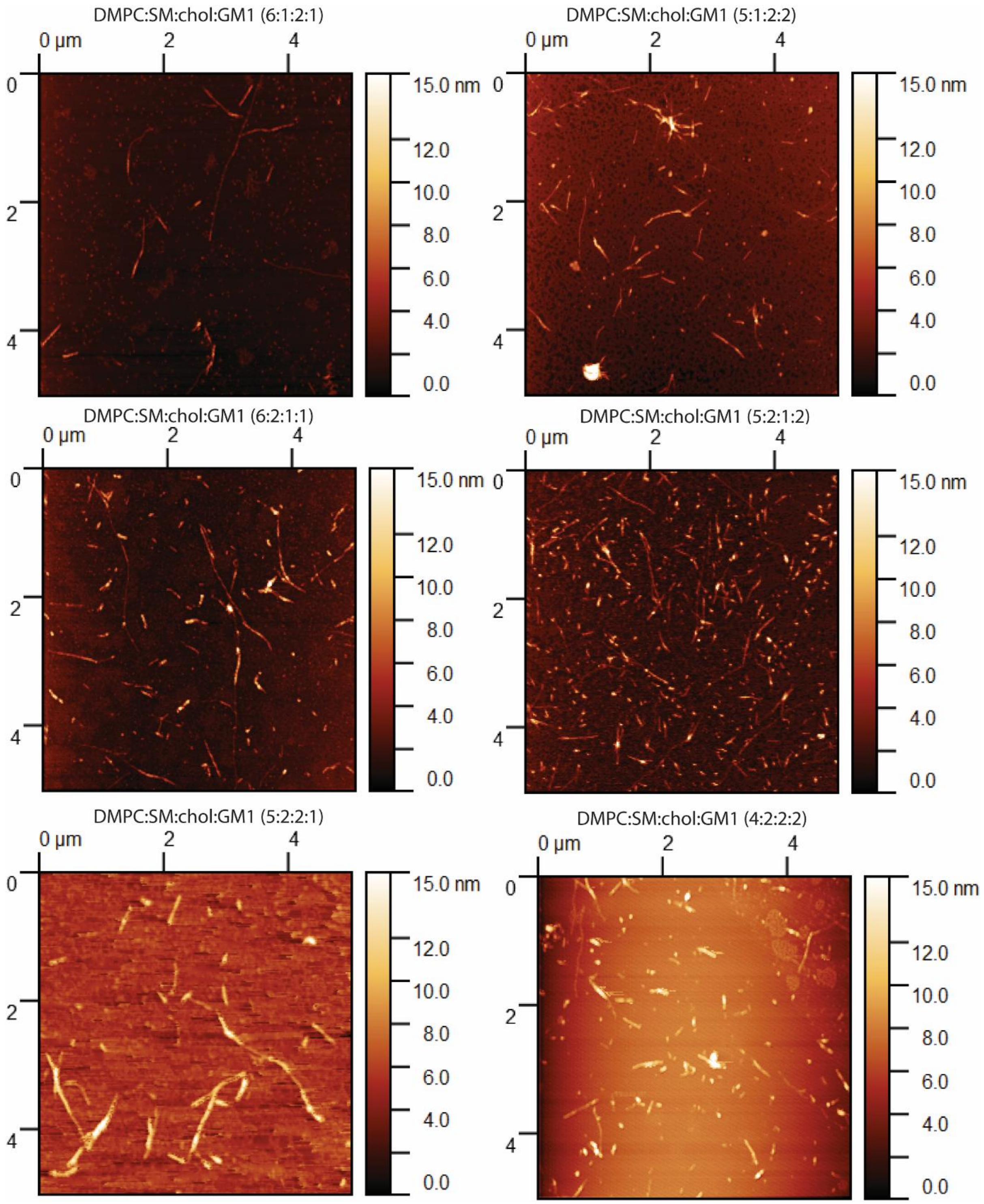

Supplementary Figure 11. AFM images of Aβ(1-42) fibrils formed in presence of quaternary LUVs. Representative AFM images of end-point Aβ(1-42) fibril samples in presence of quaternary LUV systems, underlying the data analysis shown in Supplementary Figure 12. A total of 17 images were acquired for DMPC:SM:Chol:GM1 (6:1:2:1), 14 images for (5:1:2:2), 20 images for (6:2:1:1) and (5:2:1:2), 12 images for (5:2:2:1), and 16 images for (4:2:2:2).

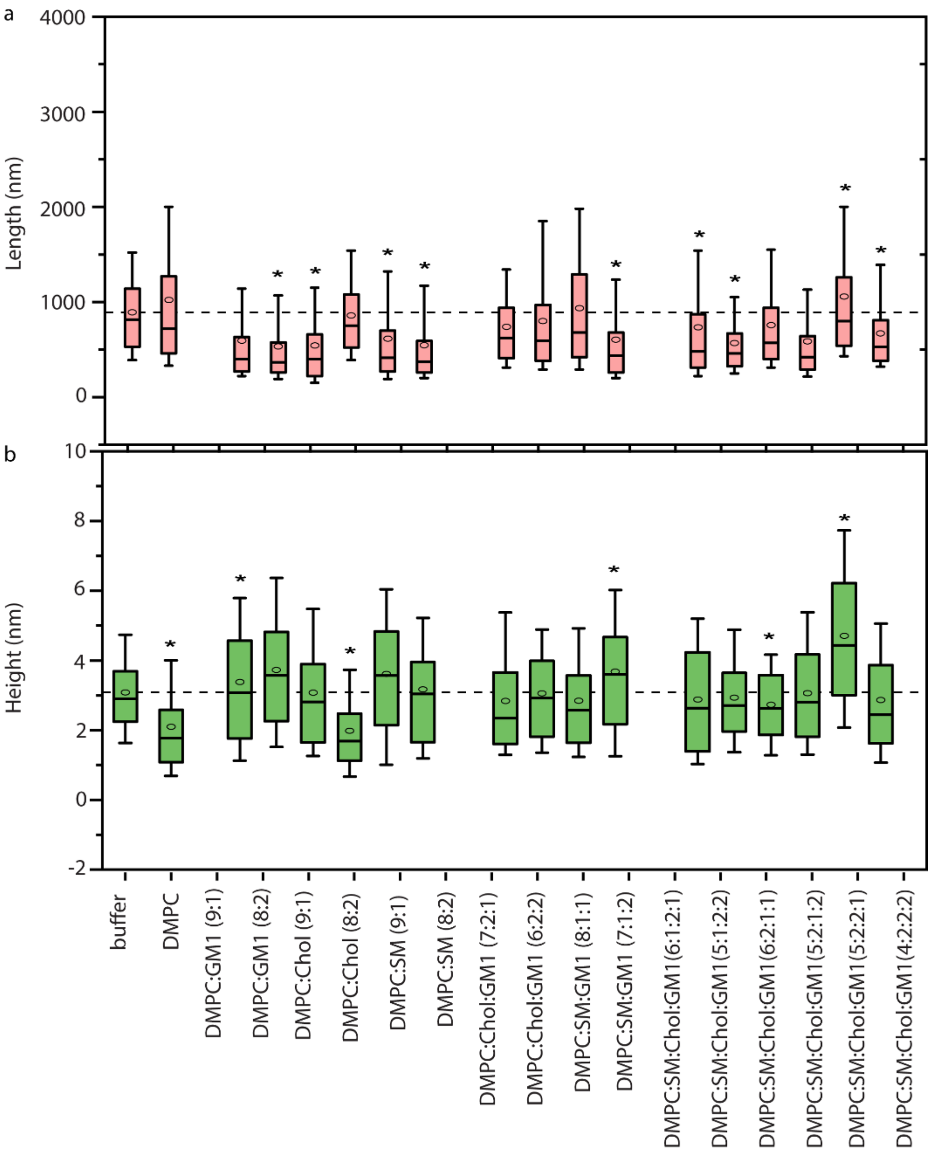

Supplementary Figure 12. Morphological characteristics of Aβ(1-42) fibrils. Measurement of length and height (as described in Methods section) of amyloid fibrils formed in the absence (buffer) and presence of different lipid vesicles extracted from AFM images of the type shown in Supplementary Figures 8-11. Each box plot represents the 25-75 percentile of measured height (**a**) or length (**b**) for 200 fibrils in each condition. Error bars indicate the range of measurements. The mean is shown as an open circle, and the median as a black line in each box. Two tailed paired sample t-test was performed to compare each aggregation condition in presence of different liposomes with aggregation in buffer, * denotes $p < 0.05$. The analysis was performed using OriginPro Software.

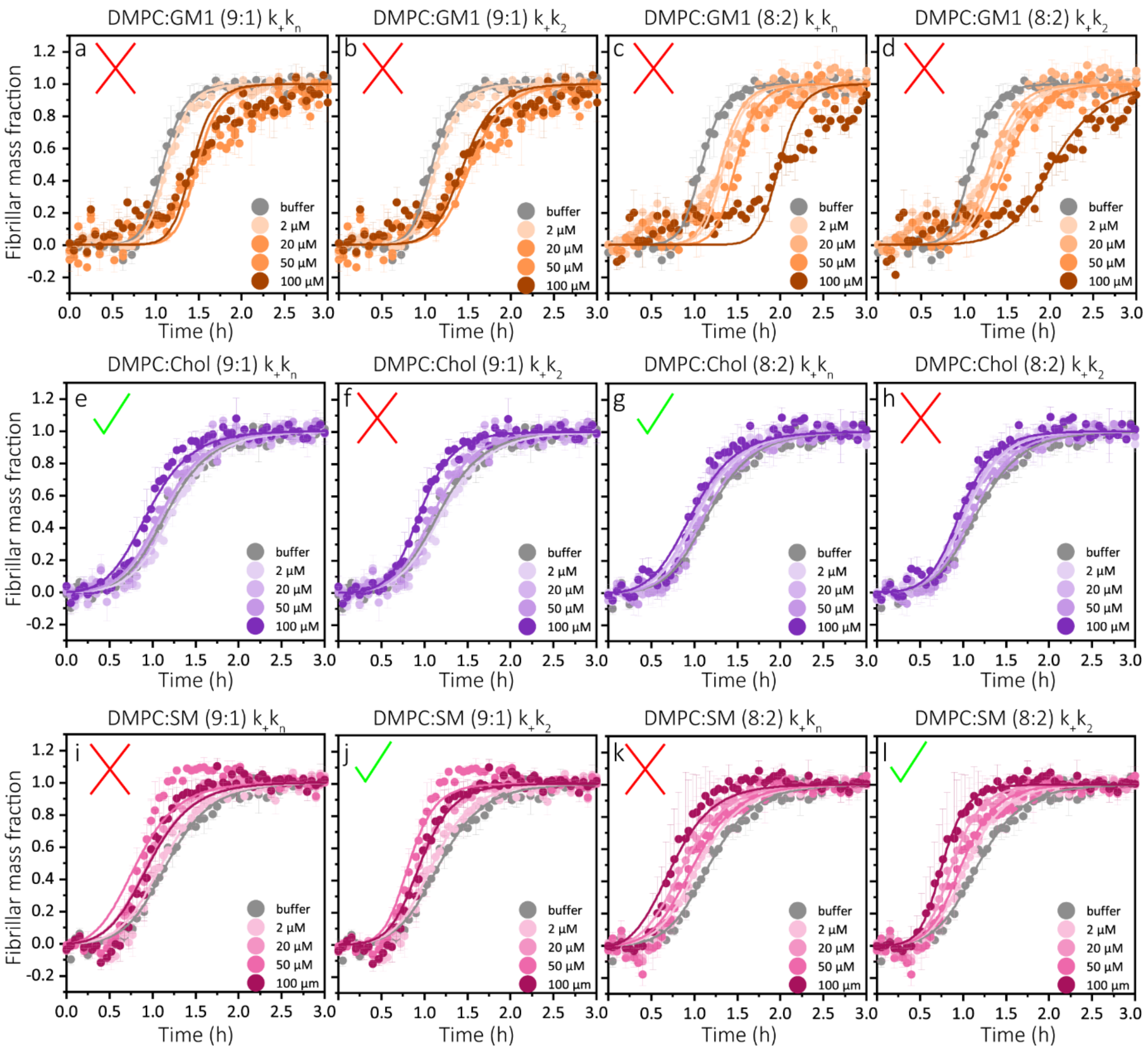

Supplementary Figure 13. Fitting of Aβ(1-42) aggregation kinetics. The figure shows fits of the aggregation kinetic data from Supplementary Figure 6 for (**a-d**) DMPC:GM1, (**e-h**) DMPC:Chol and (**i-l**) DMPC:SM vesicles to a secondary nucleation dominated aggregation model with saturation. Two fits per aggregation condition were performed, with either k_+k_n or k_+k_2 as the free (fitted) parameter. The green tick indicates the best fitting model in the pair and the red bracket the non-fitting model as evaluated by mean residual error (MRE) analysis, see Supplementary Table 2. The lipid composition and the fitted parameter are given in the heading of each graph.

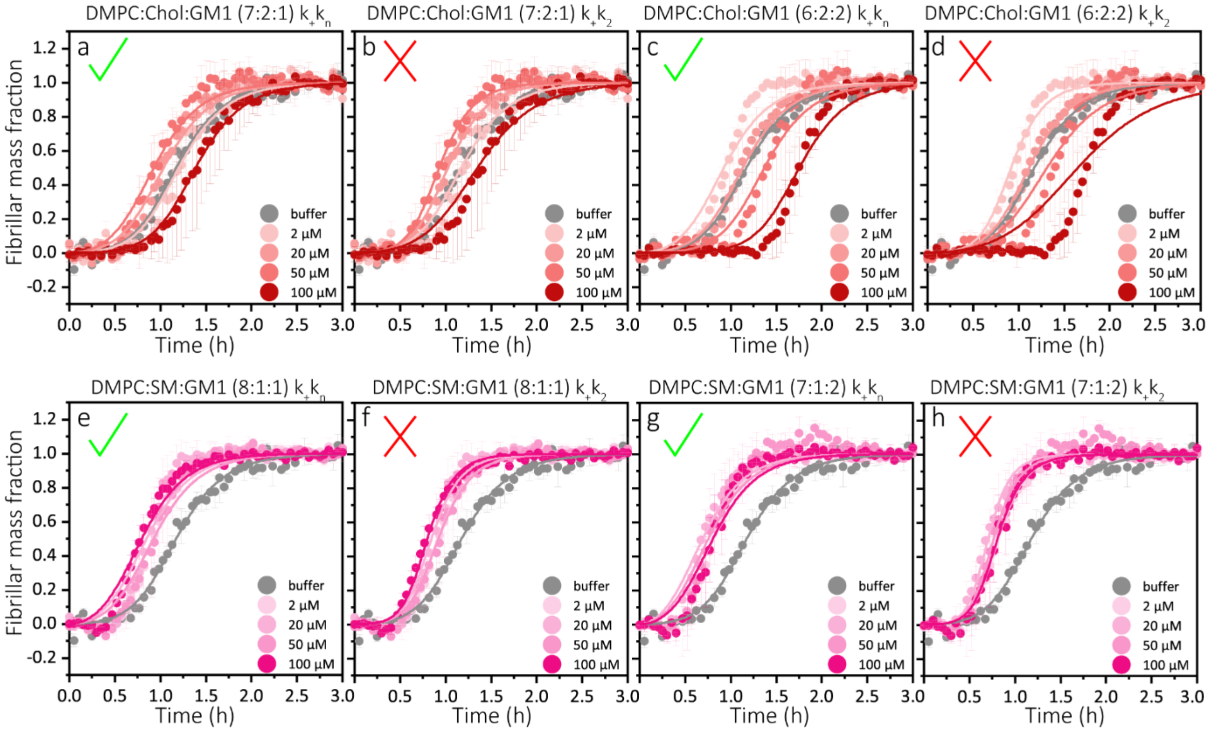

Supplementary Figure 14. Fitting of Aβ(1-42) aggregation kinetics. The figure shows fits of the aggregation kinetic data from Supplementary Figure 6 for (**a-d**) DMPC:Chol:GM1 and (**e-h**) DMPC:SM:GM1 vesicles to a secondary nucleation dominated aggregation model with saturation. Two fits per aggregation conditions were performed, with either k_+k_n or k_+k_2 as the free (fitted) parameter. The green tick indicates the best fitting model in the pair and the red bracket the non-fitting model as evaluated by mean residual error (MRE) analysis, see Supplementary Table 2. The lipid composition and the fitted parameter are given in the heading of each graph.

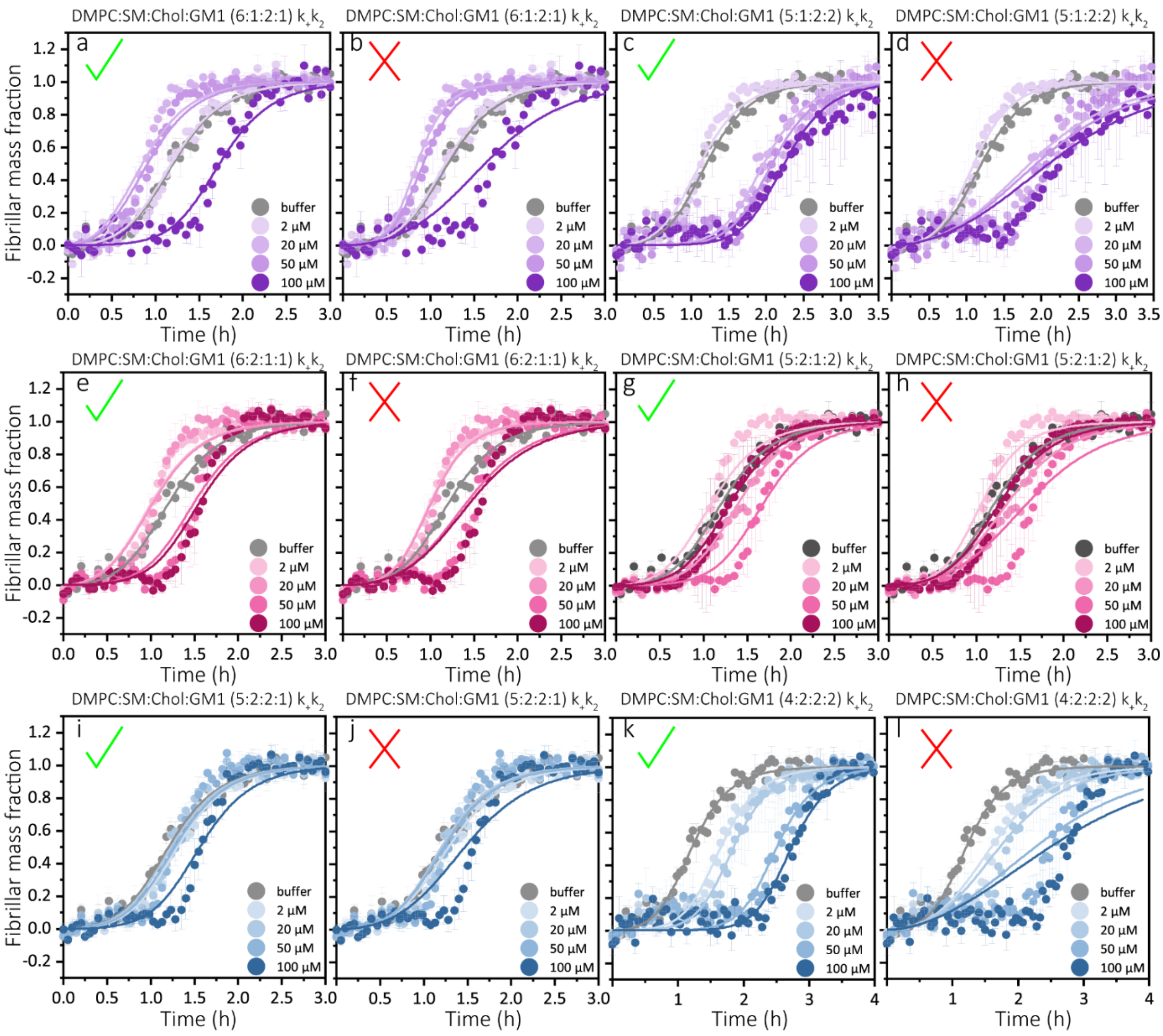

Supplementary Figure 15. Fitting of Aβ(1-42) aggregation kinetics. a-l The figure shows fits of the aggregation kinetic data from Supplementary Figure 6 for lipid vesicles with DMPC, SM, Chol and GM1 to a secondary nucleation dominated aggregation model with saturation. Two fits per aggregation condition were performed, with either $k_{+}k_{n}$ or $k_{+}k_{2}$ as the free (fitted) parameter. The green tick indicates the best fitting model in the pair and the red bracket the non-fitting model as evaluated by mean residual error (MRE) analysis, see Supplementary Table 2. The lipid composition and the fitted parameter are given in the heading of each graph.

Supplementary Table 2. Mean residual error (MRE) for Aβ(1-42) aggregation kinetic fits. MRE values of the fittings to the Aβ(1-42) aggregation kinetics data shown in Supplementary Figures 13-15. Lower MRE values indicate a better fit between the model and the experimental data.

**Type**	**Vesicle type**	**Molar Fraction**	**MRE**
Binary (Fit for k_+k_n)	DMPC:GM1	9:1	0.0182
		8:2	0.0205
	DMPC:Chol	9:1	0.00647
		8:2	0.00396
	DMPC:SM	9:1	0.0104
		8:2	0.00762
Binary (Fit for k_+k_2)	DMPC:GM1	9:1	0.0129
		8:2	0.0168
	DMPC:Chol	9:1	0.00675
		8:2	0.00401
	DMPC:SM	9:1	0.00926
		8:2	0.00701
Ternary (Fit for k_+kn)	DMPC:SM:GM1	8:1:1	0.00739
		7:1:2	0.00789
	DMPC:Chol:GM1	7:2:1	0.0129
		6:2:2	0.00980
Ternary (Fit for k_+k2)	DMPC:SM:GM1	8:1:1	0.00449
		7:1:2	0.005
	DMPC:Chol:GM1	7:2:1	0.0138
		6:2:2	0.0131
Quaternary (Fit for k_+kn)	DMPC:SM:Chol:GM1	6:1:2:1	0.00693
		5:1:2:2	0.0130
		6:2:1:1	0.00997
		5:2:1:2	0.0121
		5:2:2:1	0.00733
		4:2:2:2	0.0105
Quaternary (Fit for k_+k2)	DMPC:SM:Chol:GM1	6:1:2:1	0.00736
		5:1:2:2	0.0148
		6:2:1:1	0.0121
		5:2:1:2	0.0148
		5:2:2:1	0.00950
		4:2:2:2	0.0223

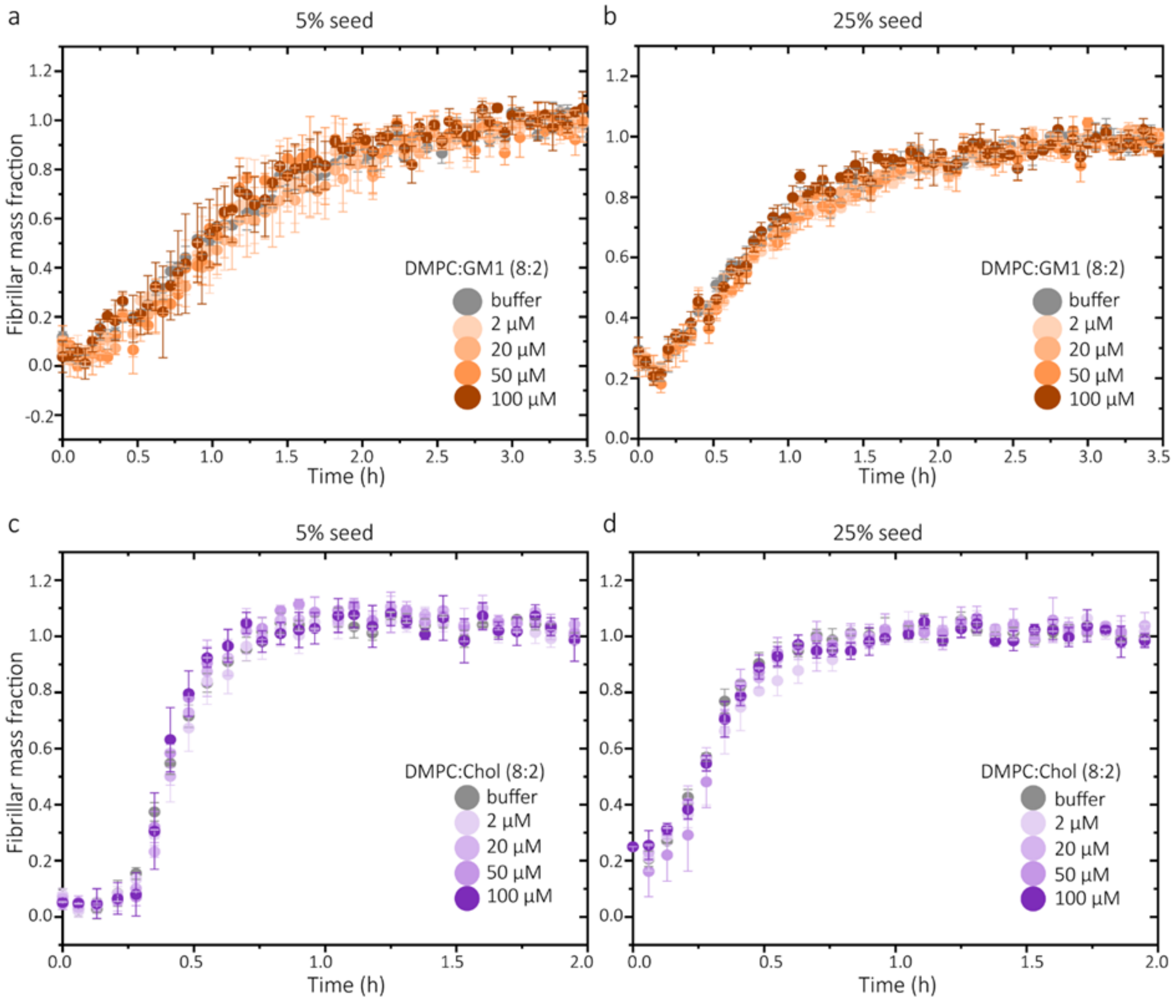

Supplementary Figure 16. Seeded aggregation kinetics of Aβ(1-42) in presence of GM1 or Chol containing binary LUVs. Normalized aggregation kinetics of 2 µM Aβ(1-42) in absence (buffer) and presence of increasing concentration of LUVs composed of DMPC:GM1 (8:2) with (**a**) 5% seeds or (**b**) 25% seeds, and DMPC:Chol (8:2) with (**c**) 5% seeds or (**d**) 25% seeds. Error bars represent the mean and standard deviation of three technical replicates (n=3).

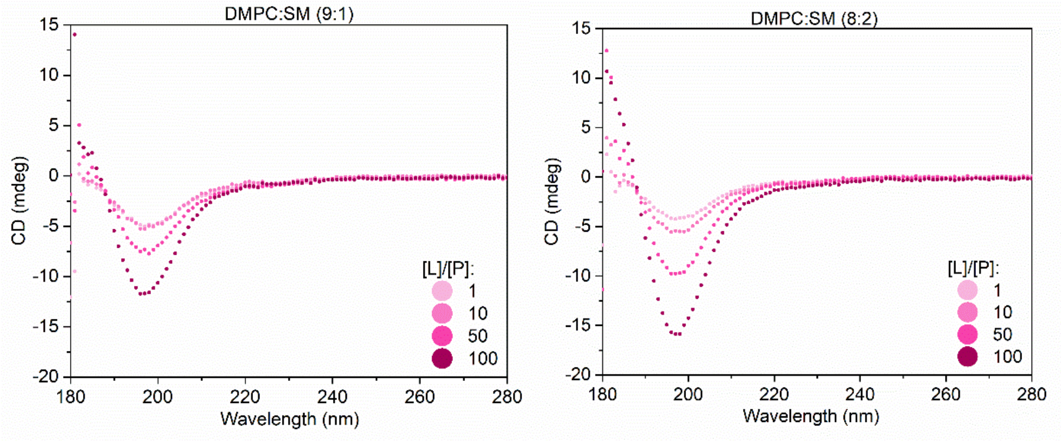

Supplementary Figure 17. **Circular dichroism (CD) spectra of Aβ(1-42) monomers in presence of SM binary LUVs.** CD spectra of a 10 µM freshly monomerized Aβ(1-42) solution (see Methods) recorded immediately upon addition of the LUVs DMPC:SM 9:1 (left) and DMPC:SM 8:2 (right) at a molar ratio, [L]/[P] of 1, 10, 50 or 100.
