# Supplementary material for: Ganglioside GM1 slows down Aβ(1-42) aggregation by a primary nucleation inhibitory mechanism that is modulated by sphingomyelin and cholesterol
Source: Commun Chem. 2025 Dec 13;9:39. doi: 10.1038/s42004-025-01846-y (PMC12824409; doi:10.1038/s42004-025-01846-y)
Supplement: Supplementary file 2 — Reporting Summary [file 42004_2025_1846_MOESM2_ESM.pdf]

nature portfolio

Corresponding author(s): DAPR COMMSCHEM-25-0629A

Last updated by author(s): Nov 21, 2025

Reporting Summary

Nature Portfolio wishes to improve the reproducibility of the work that we publish. This form provides structure for consistency and transparency in reporting. For further information on Nature Portfolio policies, see our Editorial Policies and the Editorial Policy Checklist.

Statistics

For all statistical analyses, confirm that the following items are present in the figure legend, table legend, main text, or Methods section.

n/a	Confirmed	
☐	☒	The exact sample size (*n*) for each experimental group/condition, given as a discrete number and unit of measurement
☐	☒	A statement on whether measurements were taken from distinct samples or whether the same sample was measured repeatedly
☐	☒	The statistical test(s) used AND whether they are one- or two-sided<br>*Only common tests should be described solely by name; describe more complex techniques in the Methods section.*
☐	☒	A description of all covariates tested
☐	☒	A description of any assumptions or corrections, such as tests of normality and adjustment for multiple comparisons
☐	☒	A full description of the statistical parameters including central tendency (e.g. means) or other basic estimates (e.g. regression coefficient) AND variation (e.g. standard deviation) or associated estimates of uncertainty (e.g. confidence intervals)
☒	☐	For null hypothesis testing, the test statistic (e.g. *F*, *t*, *r*) with confidence intervals, effect sizes, degrees of freedom and *P* value noted<br>*Give P values as exact values whenever suitable.*
☒	☐	For Bayesian analysis, information on the choice of priors and Markov chain Monte Carlo settings
☒	☐	For hierarchical and complex designs, identification of the appropriate level for tests and full reporting of outcomes
☐	☒	Estimates of effect sizes (e.g. Cohen's *d*, Pearson's *r*), indicating how they were calculated

Our web collection on statistics for biologists contains articles on many of the points above.

Software and code

Policy information about availability of computer code

Data collection	ThT kinetics was collected on a FluoStar OPTIMA plate reader (BMG Labtech) with OPTIMA 2.20R2 software.<br>CD spectra were collected on a Chirascan spectropolarimeter (Applied Photophysics) with Pro-Data Chirascan version 4.5.1848.0 software.<br>Laurdan fluorescence was collected on a Clariostar Plus (BMG) plate reader with CLARIOstar version 5.70 R2 software.<br>AFM images were collected on an NTEGRA Prima (NT-MDT) with Nova version 1.1.0.1820 software
Data analysis	Data was analysed using OriginPro 2024 version 10.0.0.154, Gwyddion version 2.69, and Amylofit (https://amylofit.com/amylofitmain/login/)

For manuscripts utilizing custom algorithms or software that are central to the research but not yet described in published literature, software must be made available to editors and reviewers. We strongly encourage code deposition in a community repository (e.g. GitHub). See the Nature Portfolio guidelines for submitting code & software for further information.

Data

Policy information about availability of data

All manuscripts must include a data availability statement. This statement should provide the following information, where applicable:

- Accession codes, unique identifiers, or web links for publicly available datasets
- A description of any restrictions on data availability
- For clinical datasets or third party data, please ensure that the statement adheres to our policy

Data that support the findings of this study have been deposited on figshare.com, doi: 10.6084/m9.figshare.30674660.

Research involving human participants, their data, or biological material

Policy information about studies with human participants or human data. See also policy information about sex, gender (identity/presentation), and sexual orientation and race, ethnicity and racism.

Reporting on sex and gender	Not relevant to the study
Reporting on race, ethnicity, or other socially relevant groupings	Not relevevant to the study
Population characteristics	Not relevant to the study
Recruitment	No human participants in the study
Ethics oversight	Not relevant to the study

Note that full information on the approval of the study protocol must also be provided in the manuscript.

Field-specific reporting

Please select the one below that is the best fit for your research. If you are not sure, read the appropriate sections before making your selection.

☒ Life sciences ☐ Behavioural & social sciences ☐ Ecological, evolutionary & environmental sciences

For a reference copy of the document with all sections, see nature.com/documents/nr-reporting-summary-flat.pdf

Life sciences study design

All studies must disclose on these points even when the disclosure is negative.

Sample size	ThT kinetics, laurdan assay, and cytotoxicity are recommended to be performed in technical triplicate due to measuring variance. Hence this was done. CD spectra acquisition was optimized to achieve good signal-to-noise, resulting in accumulation and averaging of five consecutive scans.
Data exclusions	No data exclusions.
Replication	All data were replicated at least three times, successfully
Randomization	This study had no relevant parameters for randomization.
Blinding	No experiment could be affected by the operator, blinding was hence not needed.

Reporting for specific materials, systems and methods

We require information from authors about some types of materials, experimental systems and methods used in many studies. Here, indicate whether each material, system or method listed is relevant to your study. If you are not sure if a list item applies to your research, read the appropriate section before selecting a response.

Materials & experimental systems

n/a	Involved in the study	
☒	☐	Antibodies
☐	☒	Eukaryotic cell lines
☒	☐	Palaeontology and archaeology
☒	☐	Animals and other organisms
☒	☐	Clinical data
☒	☐	Dual use research of concern
☒	☐	Plants

Methods

n/a	Involved in the study	
☒	☐	ChIP-seq
☒	☐	Flow cytometry
☒	☐	MRI-based neuroimaging

Eukaryotic cell lines

Policy information about cell lines and Sex and Gender in Research

Cell line source(s)	SH-SY5Y human neuroblastoma (ATCC-CRL-2266) were purchased from Sigma-Aldrich
Authentication	The SH-SY5Y cell line was authenticated by short tandem repeat (STR) analysis
Mycoplasma contamination	The SH-SY5Y cell line tested negative for mycoplasma contamination.
Commonly misidentified lines (See ICLAC register)	Not used.

Plants

Seed stocks	not applicable
Novel plant genotypes	not applicable
Authentication	not applicable
